# Integrating single-cell RNA-seq datasets with substantial batch effects

**DOI:** 10.1186/s12864-025-12126-3

**Published:** 2025-10-30

**Authors:** Karin Hrovatin, Amir Ali Moinfar, Luke Zappia, Shrey Parikh, Alejandro Tejada Lapuerta, Ben Lengerich, Manolis Kellis, Fabian J. Theis

**Affiliations:** 1 Institute of Computational Biology, Helmholtz Zentrum München, Neuherberg, Germany; 2 TUM School of Life Sciences Weihenstephan, Technical University of Munich, Freising, Germany; 3 Computer Science and Artificial Intelligence Lab, Massachusetts Institute of Technology, Cambridge, MA USA; 4 Broad Institute of MIT and Harvard, Cambridge, MA USA; 5 School of Computation, Information and Technology, Technical University of Munich, Garching, Germany

**Keywords:** Single-cell RNA sequencing (scRNA-seq), Data integration, VampPrior, Latent cycle-consistency, KL regularization strength, Adversarial learning, Benchmarking

## Abstract

Integration of single-cell RNA-sequencing (scRNA-seq) datasets is standard in scRNA-seq analysis. Nevertheless, current computational methods struggle to harmonize datasets across systems such as species, organoids and primary tissue, or different scRNA-seq protocols, including single-cell and single-nuclei. Conditional variational autoencoders (cVAE) are a popular integration method, however, existing strategies for stronger batch correction have limitations. Increasing the Kullback–Leibler divergence regularization does not improve integration and adversarial learning removes biological signals. Here, we propose sysVI, a cVAE-based method employing VampPrior and cycle-consistency constraints. We show that sysVI integrates across systems and improves biological signals for downstream interpretation of cell states and conditions.

**Supplementary Information:**

The online version contains supplementary material available at 10.1186/s12864-025-12126-3.

## Introduction

The joint analysis of multiple single-cell RNA sequencing (scRNA-seq) datasets has recently provided new insights that could not have been obtained from individual datasets. For example, the pooling of datasets generated in different studies enabled cross-condition comparisons [1, 2], population-level analysis [3, 4], and revealed evolutionary relationships between individual cell types [5]. The selection of pre-clinical models, such as organoids and animals, and the characterization of their limitations likewise rely on comparison with human tissues [6–14]. Similarly, the selection of the optimal sequencing protocols requires a comparison of datasets generated with different protocols [15, 16]. Lastly, currently arising large-scale “atlases” that are posed to serve as references of cell biology are aimed to combine public datasets with substantial technical and biological variation, including multiple organs and developmental stages [17]. Overall, with the increasing number of publicly available scRNA-seq datasets [18], the number of such cross-dataset analyses is also increasing.

These analyses can be complicated due to technical and biological differences between samples [19–21]. To overcome this, computational methods for single-cell specific data integration have been developed [22, 23] and previous benchmarks have evaluated their integration performance generally [20, 24] and on cross-species datasets specifically [19, 25]. However, the single-cell community is moving towards large-scale atlases that aim to combine a broad set of data related to a specific biological concept [14, 26] or even all-encompassing foundation models [27]. This complicates integration due to increasing data complexity and substantial batch effects. Thus, it is crucial to assess how different integration strategies perform in this setting.

**Fig. 1:**
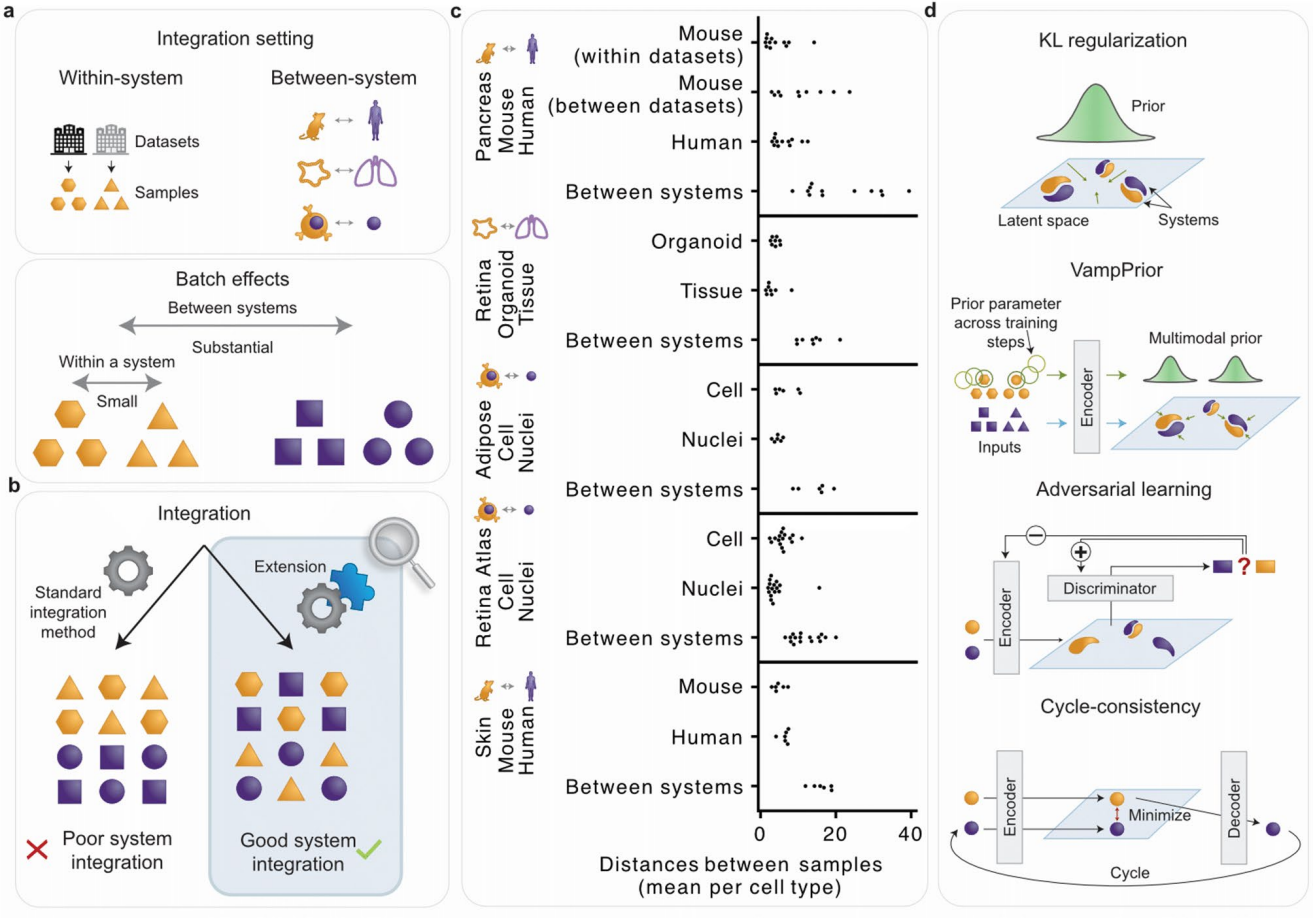
The challenge of integrating datasets with substantial batch effects. **a** Substantial batch effects are present between different biological “systems”, such as cross-species, organoid-tissue, and cell-nuclei datasets. We selected at least one dataset for each of these three between-system integration types as shown in panel **c**. **b** Integrating datasets with substantial batch effects poses a bigger challenge than integrating datasets of similar samples across laboratories, where the batch effect is smaller. In this study, we evaluate different approaches for improving cVAE-based batch correction. **c** Pre-integration distances between samples from the same or different systems. Points show mean per cell type. **d** Overview of approaches for improving integration in cVAE-based models: KL-loss-based regularization of the latent space, the use of the VampPrior as a replacement for the standard Gaussian prior, and adversarial learning and cycle-consistency loss that actively push together samples from different systems. Parts of the cVAE model were omitted from individual panels for brevity. For a detailed description of the standard cVAE and its extensions, see the methods section *Overview of cVAE-based integration approaches*

Among the most popular and best-performing methods in the past benchmarks are conditional variational autoencoder (cVAE) based models, which are able to correct non-linear batch effects, are flexible in the choice of batch covariates, and are particularly scalable to large datasets [20, 22]. Thus, they are also a method of choice for single-cell atlases [4, 28, 29]. However, while cVAE-based and other non-deep-learning methods enable good integration of batch effects caused by processing similar samples in different laboratories, they do not enable sufficient integration when differences between datasets are more substantial due to datasets originating from distinct biological or technical “systems”, such as multiple species or sequencing technologies (e.g. cell-nuclei) [19–21] (Fig. 1a, b), as demonstrated later. To enable more comprehensive single-cell atlasing efforts that will integrate diverse samples with stronger batch effects, it is thus vital to further improve the performance of the commonly used cVAE-based integration models.

Increased batch integration can be achieved via multiple extensions of cVAE models, including Kullback–Leibler divergence (KL) regularization strength tuning [30], batch distributions alignment approaches [31–33] (with latent space adversarial learning [34–36] being the prime example), and latent space cycle-consistency that was previously only used for multi-omic integration in combination with adversarial learning [37–39] (Fig. 1d). For increasing biological preservation in scRNA-seq representation learning without supervision, we previously proposed the use of the multimodal variational mixture of posteriors (VampPrior) [40] as the prior for the latent space (Fig. 1d) in a workshop paper [41], but this extension has not been explored in integration models. While prior work showed that all these strategies can be useful for single-cell data representation-learning, it is unclear how they compare to each other in the data integration setting. Thus, a systematic analysis of their strengths and weaknesses under different data integration scenarios is called for.

Here, we explore the shortcomings of popular cVAE-based integration strategies, namely KL regularization tuning and adversarial learning, and show how these can be overcome by using cycle-consistency loss and the VampPrior. In particular, we provided a detailed analysis of why individual approaches fail or outperform in diverse use cases. Furthermore, we systematically evaluate batch correction and biological preservation on cell type and sub-cell type levels, with existing [20] metrics and a newly proposed metric for assessing within-cell-type variation. We study these across three scenarios with substantial batch effects: cross-species, organoid-tissue, and cell-nuclei. These scenarios cover both substantial technical and biological confounders as well as other aspects that complicate integration and its evaluation, such as cell types with different levels of similarity across systems, multiple biological conditions, and disjoint gene feature sets. In short, we find that the combination of the VampPrior and cycle-consistency (VAMP + CYC model) improves batch correction while retaining high biological preservation, making VAMP + CYC the method of choice for integrating datasets with substantial batch effects. The VAMP + CYC model also empowers post-integration analysis of cell states and biological conditions. Our model is easily accessible to the community as part of the sciv-tools package [42] under the name sysVI, short for “integration of diverse *sys*tems with *v*ariational *i*nference”, and the here-proposed strategies could be likewise easily added to other cVAE-based integration methods.

## Results

We explored the shortcomings of existing methods for integrating substantial batch effects and subsequently developed an improved method to suit diverse use cases. We selected multiple datasets where batch effects are more substantial than observed within standard use cases that integrate samples with relatively similar biology and profiling strategies [26]. Datasets known to be challenging to integrate may include both strong biological and technical confounders [20, 26], such as multiple species, different technologies (e.g., cell-nuclei), and in vitro and in vivo samples (e.g., organoids and primary tissue) (Fig. 1a). As shown in Fig. 1c, the presence of substantial batch effects can be determined by comparing batch effect strength between samples from individual, relatively homogeneous datasets, and samples from different datasets. This can help the users to decide a priori whether a stronger batch correction will be required. Besides, post-integration evaluation can reveal insufficient batch correction, prompting the users to adapt their integration strategy [20, 26].

We included five between-system data use cases: organoids (N samples = 21, N cells = 43,505) and adult human tissue samples (N samples = 20, N cells = 54,491) of the retina, scRNA-seq (N samples = 9, N cells = 28,465) and single-nuclei RNA-seq (snRNA-seq) (N samples = 9, N nuclei = 57,599) from subcutaneous adipose tissue, scRNA-seq (N samples = 48, N cells = 265,767) and snRNA-seq (N samples = 57, N nuclei = 1,775,529) from the human retina atlas, mouse (N datasets = 8, N samples = 52, N cells = 263,140) and human (N samples = 65, N cells = 192,203) pancreatic islets, and mouse (N samples = 4, N cells = 9,816) and human (N samples = 6, N cells = 24,100) skin cells. We confirmed that in all data cases, the per-cell type distances between samples on non-integrated data are significantly smaller within systems, both within and between datasets, than between systems (Fig. 1c, Supplementary Table S1).

### Existing methods struggle with the loss of biological information when increasing batch correction

**Fig. 2:**
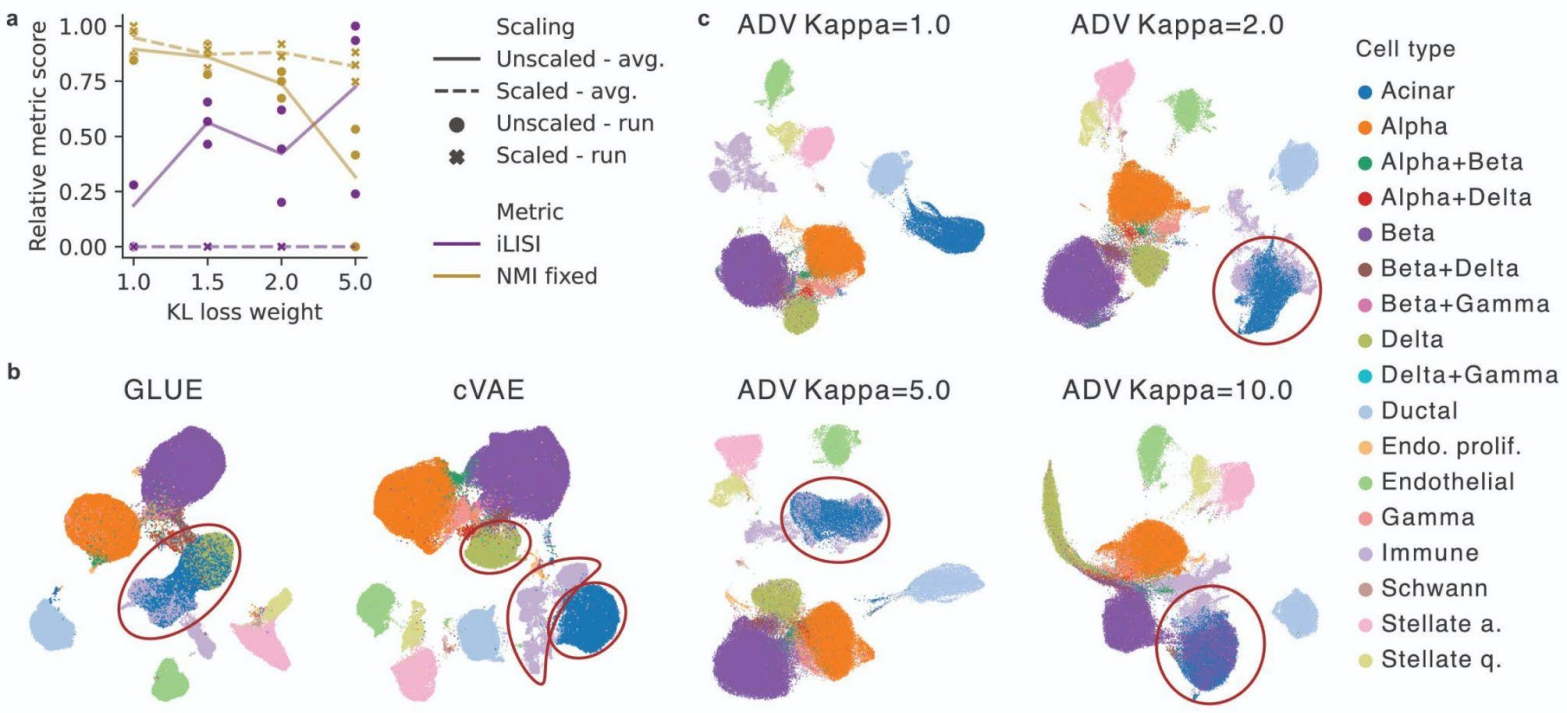
Adversarial learning and KL regularization strength tuning struggle with retaining biological variation when increasing batch correction. **a** Integration performance of scaled and unscaled cVAE embedding with different KL regularization loss weights. Shown are batch correction (iLISI, higher is better) and cell type level biological preservation (NMI fixed, higher is better) metrics scaled to [0,1] per metric. Individual runs with different seeds are shown as points and their averages (avg.) as lines. Results are presented for pancreatic mouse-human data, with similar performance trends observed in other datasets as reported in Supplementary Figure S4. **b** UMAP visualization of integrated pancreatic mouse-human data colored by cell type. Integrated representations were obtained with a standard cVAE model and the GLUE method [34], which is a well-established adversarial model and was reported to be among the best integration methods [43]. For both models, the best hyperparameter setting was used. Red circles mark examples of cell types that are mixed in the GLUE but not the cVAE integration. **c** UMAP visualization of the effect of the Kappa parameter on adversarial training. This plot shows how varying the Kappa parameter, which controls the strength of adversarial training in the ADV model, affects the integration of mouse and human pancreatic data. The data points are colored by cell type. Red circles highlight examples of cell types that are incorrectly mixed as the Kappa parameter increases

Tuning of KL regularization strength is the most widely adopted approach for tuning batch correction strength as it is part of the standard cVAE architecture. It regulates how much the cell embeddings may deviate from the standard Gaussian distribution. By definition, KL regularization does not distinguish between biological and batch information, jointly removing both of them. To assess this, we measured batch correction via graph integration local inverse Simpson’s index (iLISI) [20], which evaluates batch composition in the local neighborhoods of individual cells, and cell type level biological preservation with a modified version of normalized mutual information (NMI) [20] metric, which compares clusters from a single clustering resolution (NMI fixed) to ground-truth annotation. Indeed, increased KL regularization strength led to higher batch correction and lower biological preservation (Fig. 2a, Supplementary Figure S1). In particular, we observed that increased KL regularization strength led to some latent dimensions being set close to zero in all cells, resulting in information loss (Supplementary Figure S2). Thus, the higher batch correction score is simply a consequence of fewer embedding dimensions that were effectively used in downstream analyses. This was reflected by standard scaling of individual embedding features, resulting in the loss of KL regularization-induced changes in integration scores (Fig. 2a, Supplementary Figure S3, Supplementary Note S1). In particular, on the scaled data, higher KL regularization strength did not lead to increased batch correction or reduced biological preservation. Thus, KL weight is not a favorable approach for removing batch effects as it removes both biological and batch variation without discrimination.

The most popular cVAE extensions for actively pushing together cells from different batches are based on batch distribution alignment [31–33], especially adversarial learning [34–36, 44, 45]. However, these approaches are prone to mix embeddings of unrelated cell types with unbalanced proportions across batches. Namely, if we want to achieve indistinguishability of batches in the latent space, a cell type underrepresented in one of the systems must be mixed with a cell type present in the second system [32, 34]. We show this in a cVAE with an additional adversarial module to encourage integration of systems (ADV), and an existing single-cell integration model that leverages adversarial learning, called GLUE [34], for which it was previously shown to be among the best integration models [43]. This behavior was present across datasets, especially when increasing batch correction strength. For example, as we increased the strength of adversarial training (Kappa) in the ADV models, we saw increased mixing of acinar and immune cells, and in the most extreme cases, even beta cells (Fig. 2c). In addition, for the GLUE model, we observed mixing of delta, acinar, and immune cells in the mouse-human pancreatic dataset; astrocytes with Mueller cells in the organoid-tissue retinal dataset; and adipocyte and ASPC cells in the cell-nuclei adipose dataset (Fig. 2b, Supplementary Figure S5, Supplementary Figure S6). In contrast, these cells could be easily separated by our baseline cVAE implementation (Fig. 2b, Supplementary Figure S5). To address this issue, the authors of the GLUE method aimed to improve the adversarial loss objective by down-weighting the contribution of cells from unbalanced populations. However, this requires prior population identity knowledge, which is in GLUE initialized by a preliminary integration round. Our results show that this strategy does not guarantee good integration performance, which may be explained by biases introduced through imperfect cell cluster prior information. Therefore, adversarial learning may be problematic as a general integration strategy as datasets often have unbalanced cell population proportions due to biological differences or differences in technical protocol capture [46, 47].

### Multimodal priors and cycle-consistency for better integration

To tackle the two above-mentioned challenges of existing methods, that is the joint removal of batch and biological variation and mixing of cell populations with unbalanced proportions across systems, we propose to adapt the prior regularization of the cVAE model and add additional batch correction constraints to the general loss. In particular, we selected the VampPrior [40] and latent cycle-consistency loss [48], as they are able to better preserve biological variation.

The VampPrior was initially proposed as an alternative to standard Gaussian prior for generating more expressive latent representations due to the use of multiple prior components [40] (see methods and Fig. 1d). It was previously shown that this also applies to single-cell data [41], where it increases biological preservation, similar to other flexible priors, such as Gaussian mixtures (GM) [49–51]. Therefore, we here tested whether the VampPrior could be used to achieve a better batch correction and biological preservation tradeoff compared to a cVAE with standard Gaussian prior. We observed that while biological preservation was relatively similar in both models, the model with the VampPrior (VAMP model) had much higher batch correction performance (Supplementary Figure S1). As the VampPrior has not previously been used for batch effect removal, we investigated the cause of improved batch correction. We explored the relationship between individual prior components and cell metadata. We assigned individual cells into groups based on the prior component with the highest support for each cell and assessed whether this led to the grouping of cells by either cell types or systems. Prior component groups corresponded more strongly to cell type identity than systems (Supplementary Figure S7). This suggests that in contrast to the standard Gaussian prior that attracts cells from different systems and different cell types, using multiple priors allows individual prior components to attract cells from only a subset of cell types that nevertheless originate from both systems, which could explain the improved batch correction at a similar biological preservation score.

To test if batch correction is improved due to the multimodal nature of the VampPrior or its other properties, we compared to a VampPrior model with only one prior component that resembles a normal cVAE, but in contrast to cVAE the prior parameters are learned, and a GM prior model (GMM) as a simpler multimodal prior alternative where prior and posterior distributions are not coupled. We also tested if trainable prior components are key for integration by fixing prior component parameters and inspected if the identity of cells used for pseudoinputs initialization affects the performance.

**Fig. 3:**
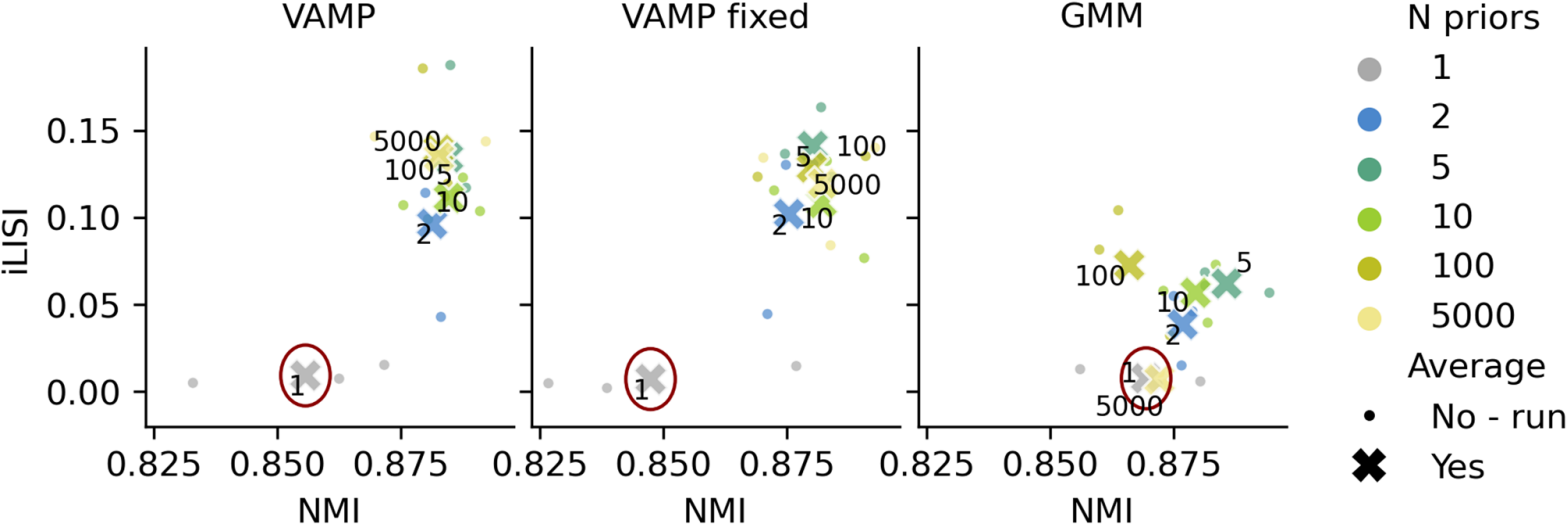
Multimodal priors improve batch correction and biological preservation. Scatter plots of cell type level biological preservation metric (NMI, higher is better) and batch correction metric (iLISI, higher is better) scores for different models (panels), including a VampPrior model with fixed prior components. Small circles represent individual runs with different seeds and crosses their averages. Points are colored by the number of prior components. The average performances of the models with a single prior component are encircled. Results are shown for the pancreatic mouse-human data, with other datasets exhibiting similar trends as presented in Supplementary Figure S8

Across integration datasets, we find that the batch correction was lower when using only one prior component, compared to two or more for both the VAMP and GMM models (Fig. 3, Supplementary Figure S8), with the one-component version being more similar to cVAE with standard normal prior (Supplementary Figure S1). However, the performance of GMM, but not VAMP, dropped again at very high prior component numbers (*N* = 5000). Therefore, even a relatively small number of prior components (e.g. five) enables good integration while keeping the number of model parameters low. Fixing of prior components had little effect on VAMP performance (Fig. 3, Supplementary Figure S8, Supplementary Note S2) and likewise, the initialization of prior components with cells from a single cell type or system did not affect performance (Supplementary Figure S9, Supplementary Note S2). This indicates that the number of prior components is key for improved batch correction. Nevertheless, VAMP outperformed GMM both in the achieved batch correction as well as in the robustness to a varying number of prior components, underlining the importance of coupling the prior and the posterior.

We propose latent cycle-consistency loss as an alternative to adversarial learning. The cycle-consistency loss directly compares only cells with an identical biological background, that is a cell and its counterfactual representation from another system (Fig. 1d). Thus, biologically distinct cell populations are not forced to overlap, unlike in models that aim to align whole system distributions in a cell identity-agnostic manner. While previous publications already used cycle-consistency for integration across modalities (e.g. scRNA-seq and scATAC-seq) [37, 39], they always combined it with adversarial learning, thus overlooking the potential benefits of using cycle-consistency alone.

**Fig. 4:**
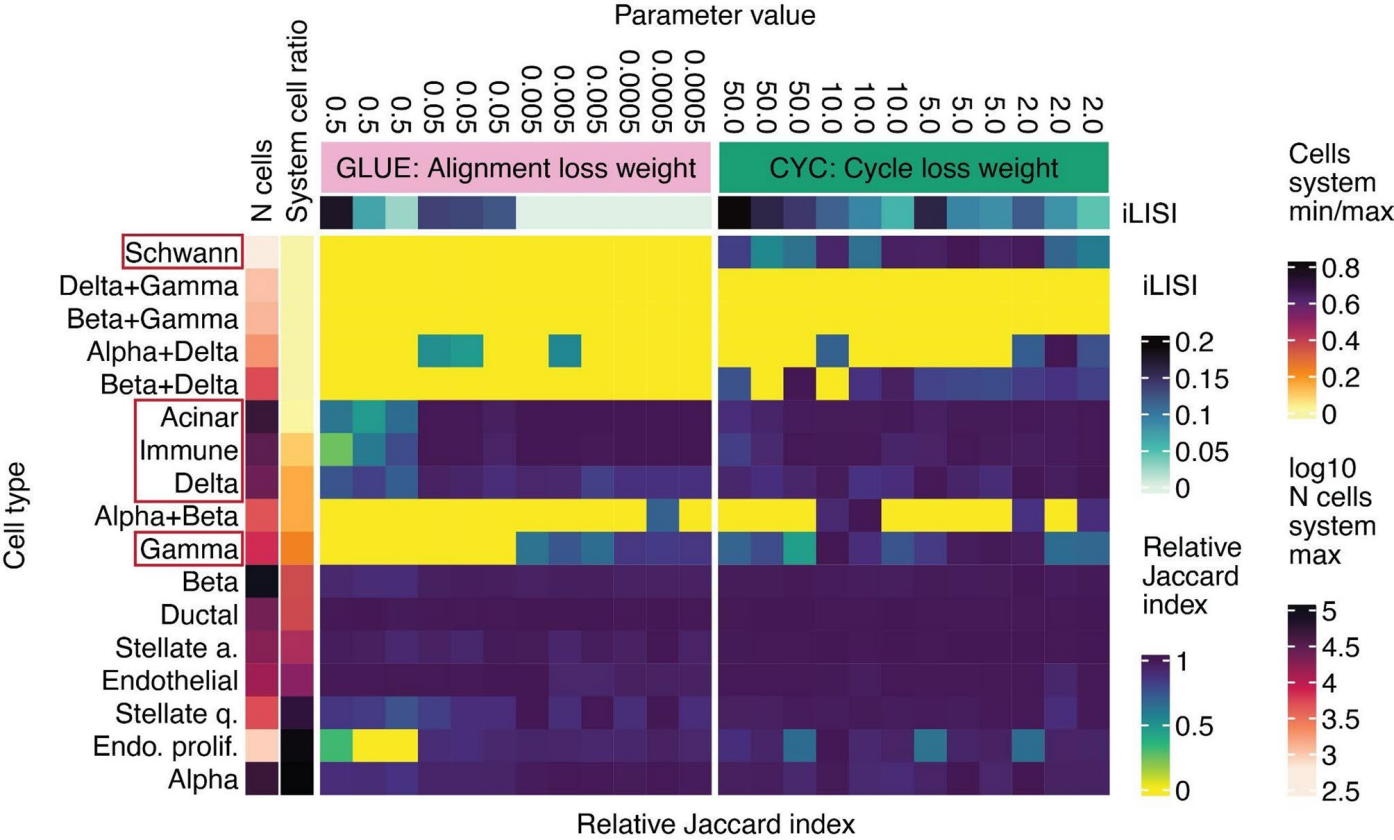
Adversarial learning is more prone to cell-type mixing than cycle-consistency loss. Shown is a cell type mixing score measured with the Jaccard index between clusters and ground-truth labels (higher is better) and iLISI score. The Jaccard index was min-max-scaled per cell type. Results are presented for integration of the pancreatic mouse-human dataset with the adversarial GLUE model and the CYC model. For both models, different weights of losses that regulate batch correction were tested. Individual cell types are annotated with the number of cells in the more abundant system and the ratio of cells between the less and the more abundant system. Red boxes mark example cell types that are commonly mixed by the adversarial model, especially when increasing batch correction strength. Cell populations representing technical doublets are marked with “+” in their name. Similar results for other datasets are shown in Supplementary Figure S6

We validated our hypothesis about cycle-consistency outperforming adversarial learning in the presence of cell populations with unbalanced proportions across systems by implementing a cVAE model with added cycle-consistency loss (CYC model) and comparing it to adversarial model GLUE. For both models, we gradually increased the weight of the loss responsible for batch correction and measured cell type preservation by comparing prior annotation and post-integration clusters with the Jaccard index. When increasing batch correction strength, we observed multiple cell types whose clusters had a lower Jaccard index in the GLUE than the CYC model, thus being merged with other cell types in GLUE (Fig. 4, Supplementary Figure S5, Supplementary Figure S6). Examples are acinar cells mixed with immune cells in the pancreatic mouse-human data, which are not biologically related, adipocytes mixed with adipose stem and progenitor cells (ASPC) in the adipose cell-nuclei data, and two glial populations (astrocytes and Mueller cells) in retinal organoid-tissue data. However, some cell types were hard to distinguish for both models, regardless of integration strength. In the pancreatic mouse-human data, these corresponded to technical doublets, which can be explained by their similarity to individual contributing cell types. Similarly, in the cell-nuclei data neutrophils co-localized with monocytes, likely due to their biological similarity, with both of them being immune cells (Supplementary Figure S5). Therefore, none of the models exhibited perfect cell type resolution based on the Jaccard index metric, which is expected as highly similar cell types often require more detailed analysis, including subclustering, for their annotation [52]. Nevertheless, the CYC model was less prone to mix both related and unrelated cell types than GLUE.

### Integration with the VampPrior and cycle-consistency offers a good tradeoff between batch correction and biological preservation

**Fig. 5:**
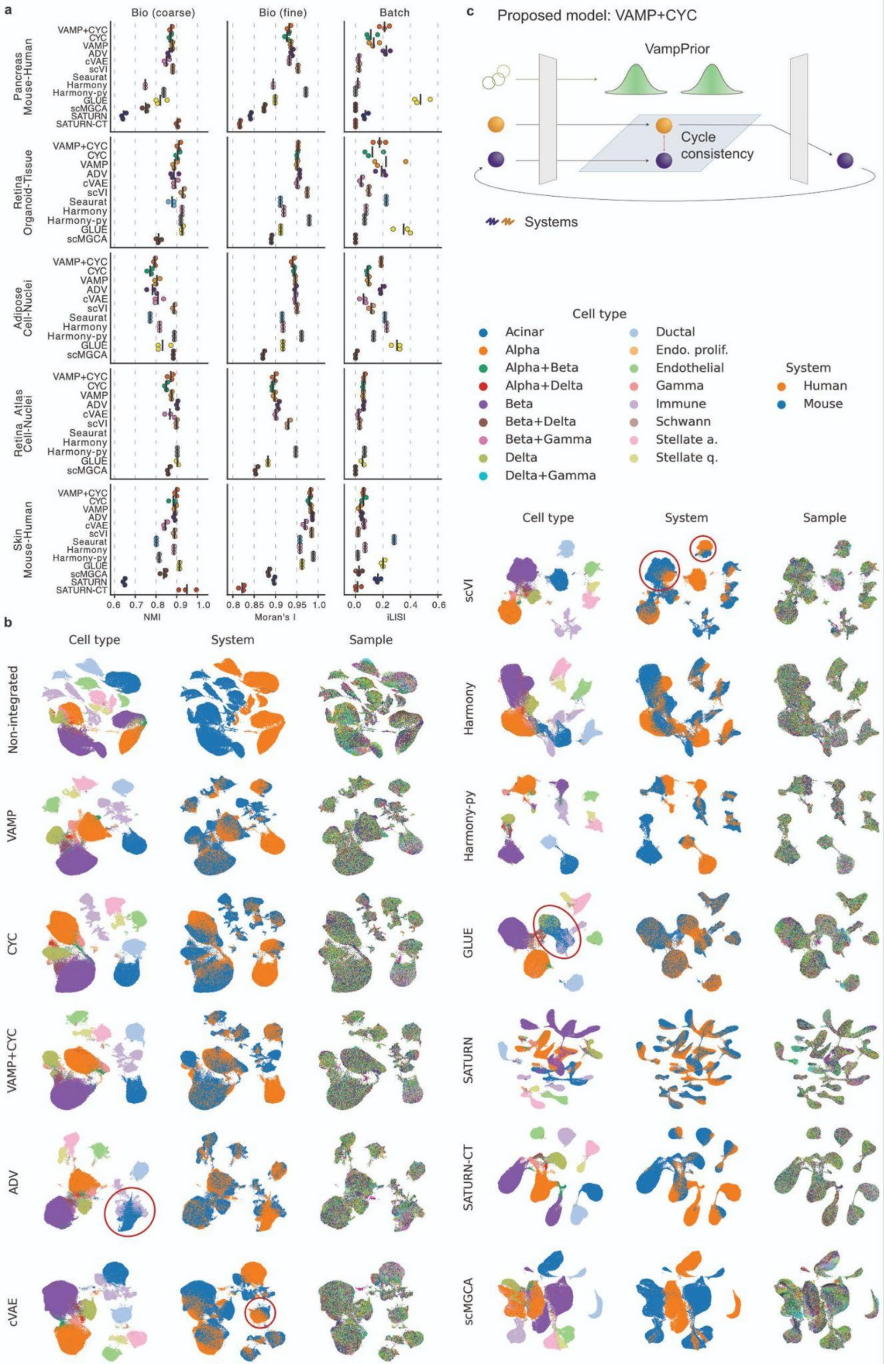
While existing models struggle with over- or under-integration, the VAMP + CYC model achieves a good trade-off between batch correction and biological preservation. **a** We show the integration performance of individual models using the best hyperparameter settings, with different integration metrics in columns (larger values indicate better integration performance) and cross-system data cases in rows, showing the average performance (vertical line) of three runs (dots). The results of all hyperparameter settings are in Supplementary Figure S1. **b** UMAPs of representative runs for the best hyperparameter setting in the pancreatic mouse-human dataset. A legend for sample colors is not shown due to the large number of samples. UMAPs for the other datasets are shown in Supplementary Figure S5. **c** Schematic representation of the VAMP + CYC model in accordance with the explanation of the VampPrior and latent cycle-consistency loss from Fig. 1d

Our analysis showed that both the VampPrior and latent cycle-consistency loss improved integration performance. Moreover, both approaches are complementary, with VampPrior providing a more expressive latent space than standard Gaussian prior and cycle-consistency loss pushing together batches without incurring cell population mixing in contrast to adversarial learning. Therefore, we propose a new integration approach that combines the two (VAMP + CYC, Fig. 5c).

We compared VAMP + CYC against ablated versions VAMP, CYC, and a cVAE baseline. All these models are based on the same underlying cVAE model with the addition of the VampPrior (VAMP), cycle-consistency loss (CYC), or their combination (VAMP + CYC). To this, we also added two established autoencoder-based models: scVI [23], which is a cVAE-based model that models raw expression counts and is among the most popular integration methods, and GLUE, which is an example of a model that uses adversarial learning. We also implemented an adversarial cVAE baseline (ADV) to individually study the effect of adversarial learning on mixing cell types across systems. For cross-species integration examples, we included SATURN [53] as a baseline. The comparison was further extended with three additional established models that do not use the autoencoder architecture: Seurat [54, 55] based on integration using canonical correlation analysis, scMGCA [56] as a graph-based [57] integration method, and Harmony [58], for which we included both implementations in R (referred to as Harmony) and Python (referred to as Harmony-py). To enable a fair comparison, we tuned model hyperparameters that directly regulate batch correction for all methods, as described in the methods. Our benchmark thus aims to depict the best possible performance of every method optimized for individual datasets (Supplementary Figure S1). Every model was run three times with different random seeds to capture random variation in model performance.

To complement our integration evaluation via iLISI and NMI, we proposed an additional metric, Moran’s I, aimed to assess fine biological preservation on the sub-cell-type level via preservation of gene expression patterns after integration. Evaluating the fine variation is of special importance as many downstream questions rely on within-cell-type population shifts that arise due to different biological conditions, such as in health and disease. We further show why the preservation of fine-grained biological variation is crucial for common biological analyses in the section *High-quality integration is key for downstream biological interpretation*.

The cVAE-based model scVI [23], which is regarded as a state-of-the-art model for scRNA-seq integration [20], consistently had excellent biological preservation (Fig. 5a), in many cases significantly higher than other autoencoder-based models (Supplementary Table S2). However, it had a relatively low batch correction compared to other models, including GLUE and our newly proposed model VAMP + CYC (Fig. 5a), especially in the pancreatic mouse-human and retinal organoid-tissue datasets that had the most substantial batch effects (Supplementary Figure S10). For example, many of the cell types, including beta and ductal cells, were also visually separated between the systems on the UMAP (Fig. 5b).

GLUE had the highest batch correction overall (Fig. 5a), which was in most cases significantly higher than other models (Supplementary Table S2), and it also had relatively good cell type preservation (NMI). However, as described above, in unbalanced datasets GLUE led to the mixing of unrelated cell types, such as delta, acinar, and immune cells in the pancreatic data (Fig. 5b). It also had lower performance on finer within-cell-type variation (Moran’s I) (Fig. 5a), which was in all cases significantly lower than the state-of-the-art model scVI as well as VAMP + CYC (Supplementary Table S2). As we saw at the cell type level, poor preservation of within-cell-type variation could be partially explained by the mixing of cell states with unbalanced proportions across systems. Thus, it is likely that the high batch correction of GLUE is not biologically meaningful as it comes at the cost of mixing unrelated cell populations. Integration with adversarial learning is thus less suited for downstream cell state analyses.

Seurat consistently localized among models with high batch correction. However, it often had relatively low cell-type preservation (NMI) and consistently low within-cell-type preservation (Moran’s I), similar to GLUE. While reducing its *k.anchor* parameter could somewhat improve cell-type preservation at the cost of batch correction, it could not rescue the poor within-cell-type preservation (Supplementary Figure S1). Furthermore, Seurat can not be used for larger datasets due to the extensive training time required (over 20 h). This makes Seurat an overall unfavorable choice.

When analyzing the two implementations of the Harmony method, we observed striking differences. Not only was the Harmony-py better able to scale to large datasets than the original R implementation, as expected, but also it had consistently higher fine within-cell-type preservation (Moran’s I). In this metric it was among the top methods, sometimes even outperforming scVI. However, this came at the cost of poor batch correction, similar to scVI. For example, in the pancreatic dataset the mouse and human beta cells resided in distinct UMAP regions, not even localising nearby each other (Fig. 5b). The only exception was the retina atlas cell-nuclei dataset, where all methods had a similar, visually good (Supplementary Figure S5c), batch correction and here Harmony-py was actually among best-performing methods. Despite good within-cell-type biological preservation, Harmony-py had relatively low cell-type preservation on a few datasets, below the level of scVI. Nevertheless, Harmony-py may be an alternative to scVI and a viable choice for datasets with less challenging batch effects.

Furthermore, while Harmony implemented in R had lower fine within-cell-type preservation than Harmony-py, this did not translate to good batch correction performance. Namely, Harmony in R varied strongly in the batch correction metric, from relatively poor to relatively good results depending on the dataset. The Harmony from R may be an inconvenient choice due to variable integration performance and extensive training time required.

The scMGCA model was less effective at batch correction than other methods, resulting in only partial mixing of cells across most datasets. Moreover, it maintained a moderate level of biological preservation, suggesting it may be more suitable for applications where the batch effect is not substantial, such as the batch effect across donors within the same experimental setup.

Overall, the existing methods can be separated into two classes: those that have generally good preservation of biological variation, also on within-cell-type level, but poor batch correction (scVI, Harmony-py, and scMGCA) and those that have strong batch-correction but are unable to preserve fine-grained biological variation (GLUE, Seurat). Thus, there is a need for methods that enable sufficient batch correction, even for datasets with substantial batch effects, while preserving both cell type and within-cell-type biological variation [20].

The VAMP + CYC model was able to achieve a better tradeoff between batch correction and biological preservation (Fig. 5a**)**. We first show that it consistently outperformed the baseline cVAE, indicating that the use of cycle-consistency loss and the VampPrior significantly improve cVAE-based integration. Specifically, the VAMP + CYC model had significantly higher batch correction than the cVAE baseline across most of the datasets (adjusted p-values < 0.1, Supplementary Table S2) while retaining comparable biological preservation in the majority of comparisons. For example, on the pancreatic mouse-human dataset cVAE was unable to batch-correct some cell types, such as beta and ductal cells, while VAMP + CYC had much stronger mixing between mouse and human cells for both of them (Fig. 5b). The only exception was the skin datasets where an extremely high KL loss weight was selected for cVAE. While this resulted in cVAE-based batch correction comparable to VAMP + CYC, the biological preservation of cVAE was significantly worse (Fig. 5a, Supplementary Figure S1, Supplementary Table S2). The VAMP + CYC model was also able to achieve higher batch correction at comparable biological preservation than the individual CYC and VAMP models (Supplementary Figure S1), supporting the combined use of both cycle-consistency loss and the VampPrior. Namely, the selected VAMP + CYC model had significantly higher batch correction than the two ablated models in the pancreatic mouse-human and adipose cell-nuclei datasets (Supplementary Table S2).

In comparison to GLUE and Seurat, the VAMP + CYC model showed lower batch correction but had higher fine-grained biological preservation in all datasets (Fig. 5a). Moreover, unlike GLUE, it was not prone to mixing of cell types with different proportions across systems, as described above. On the other hand, VAMP + CYC had somewhat lower fine-grained biological preservation than scVI and Harmony-py, but had in almost all datasets much better batch correction. Thus, the VAMP + CYC model seems to be better balanced than existing models as it has relatively good performance in both biological preservation and batch correction. It thus fills a gap between groups of models that tend to over-integrate (GLUE and Seurat) or under-integrate (scVI, Harmony-py, and scMGCA). This recommends VAMP + CYC for integrating datasets with substantial batch effects. However, as metric scores alone cannot fully assess biological usefulness of a model [26], we also analysed how different models affect downstream biological interpretation in the section *High-quality integration is key for downstream biological interpretation*.

Furthermore, we believe that the VAMP + CYC model will be convenient to use in the future. The fine-tuning of the integration hyperparameters led to similar performance characteristics in all datasets (Supplementary Figure S1), easing the choice of the ideal hyperparameter values. Moreover, VAMP + CYC is scalable to large datasets, requiring less than 50 min for half a million cells and memory comparable with scVI (Supplementary Figure S19). While its memory requirements are slightly higher than scVI model and its runtime is double compared to scVI model, likely originating from additional costs related to the VampPrior and cycle-consistency loss computation, it was nevertheless able to integrate even the largest retina atlas dataset with two million cells.

### Models specific for cross-species integration do not outperform VAMP + CYC

Cross-species integration is complicated due to different gene sets across species. Thus, we further assessed models that accept different input genes across systems, namely GLUE and SATURN [53], which is an example of a model designed specifically for cross-species integration. We ran both models with one-to-one orthologues (OTO) as well as with a flexible orthology (FO) gene set that included different types of orthologues and non-orthologous genes on the pancreatic mouse-human dataset. Integration with FO input genes did not outperform the OTO approach in either GLUE or SATURN (Supplementary Figure S1). Furthermore, despite SATURN using protein embeddings to improve gene linking across species, it had significantly lower batch correction than either VAMP + CYC or GLUE (Fig. 5a, adjusted p-values < 0.05, Supplementary Table S2). Overall, this indicates that a more advanced gene mapping may not be needed for mouse-human integration, as VAMP + CYC was able to outperform even dedicated cross-species integration methods. However, for more divergent species or species with lower-quality genome annotation and orthology information, using FO genes could still be beneficial [19].

As SATURN requires prior cell type labels for every system, we ran it with clustering-based labels to make it comparable to other models that are likewise not given prior cell type information, which is in practice often not available. Besides that, we also added a version with ground-truth cell-type labels (SATURN-CT). However, using ground-truth labels may lead to a positive bias in the NMI metric. Both SATURN and SATURN-CT performed poorly in biological preservation, with SATURN-CT having a bias towards higher NMI scores while retaining low Moran’s I (Fig. 5a). The poor preservation of within-cell-type structure was also evident from UMAPs. For example, the numerous immune subclusters observed on the VAMP + CYC embedding were not present after integration with SATURN and SATURN-CT (Fig. 5b). The poor biological preservation can be explained by over-reliance on prior cell cluster labels in the contrastive learning objective, which pushes all cells with the same label together, thus neglecting within-cell-type variation (Supplementary Note S3).

### High-quality integration is key for downstream biological interpretation

To ensure that a model is of good quality, it must not only have relatively high integration metrics, but it must also be able to provide meaningful biological interpretations. Therefore, we used the integrated embeddings of the evaluated methods for multiple downstream tasks, including the discovery of molecular heterogeneity within individual cell types as well as cross-system comparison of cell types and conditions.

An important characteristic of integration is to preserve within-sample variation, which is not related to batch effects. This is key for studying different cell states that arise due to cell specialization or spatial niches. This is also an integral part of harmonizing fine-grained cell state annotation, which is one of the main aims of the integration and atlas-building projects. In particular, while coarse cell types often have established markers that can be used for annotation, the more specialized cell states are often inconsistently annotated across datasets and must be re-defined based on multi-dataset consensus [1, 26]. However, fine-grained annotation is not possible if such variation is lost after integration.

**Fig. 6:**
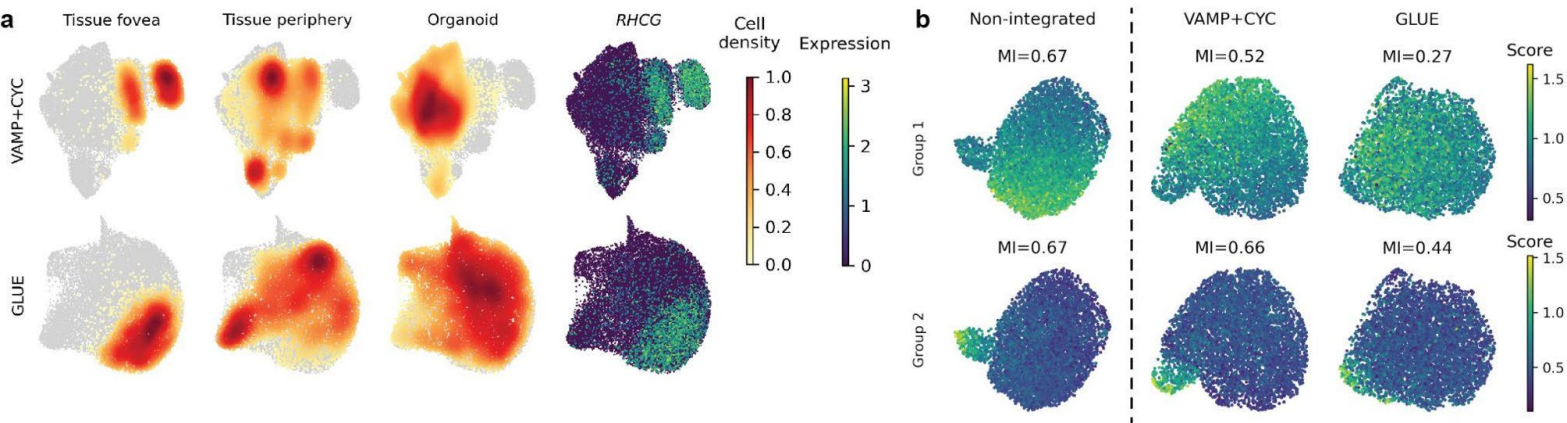
VAMP + CYC empowers post-integration analysis of biological variation. **a** UMAPs of Mueller cells based on representative runs for the best hyperparameter setting in the organoid-tissue dataset. The UMAPs are colored by the cell density of organoid and tissue periphery cells, which are expected to overlap, and of tissue fovea cells expressing *RHCG*, which are expected to separate from the other two cell populations. Results for other methods are shown in Supplementary Figure S11. **b** Expression of gene groups known to be heterogeneous within individuals, shown on UMAPs of beta cells from one healthy adult mouse pancreatic sample from the pancreatic mouse-human data. We compare the heterogeneity before and after integration and quantitatively assess gene group variability with Moran’s I (MI). For every model, one representative run from the best hyperparameter setting was selected (as shown in Fig. 5b). Results for all models and the remaining gene groups are shown in Supplementary Figure S12c

We examined the preservation of known cell states on mouse pancreatic beta cells, for which within-sample heterogeneity in the expression of multiple gene programs has been previously reported [1]. This heterogeneity was much better preserved by VAMP + CYC than GLUE (Fig. 6b, Supplementary Figure S12c) and was similar to the heterogeneity observed in the non-integrated data. While some other methods also retained gene program heterogeneity (e.g., scVI and Harmony-py), they did not integrate beta cells between mouse and human data (Fig. 5b), rendering harmonization of fine-grained cell states across species impossible. Therefore, as VAMP + CYC has low within-system information loss and strong batch correction, it is best suited for post-integration analysis of cell states.

Direct comparison of systems on the integrated embedding requires striking a balance between under and over-integration, that is, to align only biologically related, but not biologically distinct cells. To achieve this, the model’s objective must also reflect this balance by jointly optimizing batch correction and biological preservation. An example is scGEN [59] that fails to correctly align biological populations (Supplementary Note S4). This is key for diverse downstream use cases. For example, when working with models of human biology in vitro (e.g., organoids) or in vivo (e.g., different species), it is important to understand which cell types correspond to their human counterparts and which are more biologically distinct. Furthermore, when employing disease models, it is important to understand if their disease effects differ from the healthy state in a similar fashion as in humans.

Here, we assess how well models achieve this balance by inspecting individual cases where systems should, in fact, not be completely integrated due to biological differences. We saw that all evaluated models correctly separated retinal pigment epithelial cells between organoid and tissue data [7], with the two clusters being the most proximal in Seurat integration (Supplementary Figure S5a). This indicates that strong biological differences are preserved after integration.

Moreover, we assessed the preservation of finer cross-system biological differences, where cell states within cell types are slightly shifted between biological conditions. We show this on Mueller cells, for which it was reported that *RHCG*-expressing fovea cells from primary tissue are distinct from the tissue periphery and organoid cells [7]. This more subtle biological variation was lost in some models, such as GLUE and Seurat, leading to the co-localization of cells from all three populations (Fig. 6a, Supplementary Figure S11, Supplementary Figure S17a). On the other hand, scVI, which has weaker integration, failed to align tissue periphery and organoid cells. Performance similar to scVI was also observed for Harmony-py. In contrast, VAMP + CYC correctly aligned Mueller cells from tissue periphery and organoid samples while separating cells from tissue fovea samples.

However, as discussed above, GLUE is particularly sensitive to the imbalance of datasets across systems. This may be a reason for its under-performance in the retinal dataset where the cells from tissue fovea do not have a corresponding population in the organoid dataset. Thus, we performed a similar analysis on the skin dataset that contains two corresponding conditions (healthy and diseased) in both systems (mouse and human). The mouse and human skin datasets both contained disease-related inflammatory fibroblasts originating from recessive dystrophic epidermolysis bullosa [60] and psoriasis [61], respectively. This inflammatory phenotype distinguishes disease-related and healthy fibroblasts.

In this dataset, GLUE performed the best, more strongly aligning the human diseased cells with the mouse diseased cells than the healthy cells (Supplementary Figure S17e). While this was also observed in VAMP + CYC, the ratio between the two was less pronounced. In contrast, scVI and Harmony almost equally mixed the human diseased cells with both mouse healthy and diseased cells, being unable to correctly distinguish between biological and technical variation. Moreover, this issue was even more substantial for Seurat, which had a somewhat stronger mixing between human diseased and mouse healthy cells than mouse diseased cells. This indicates that GLUE can be the method of choice when integrating well-balanced data, with VAMP + CYC following.

Interestingly, most methods had a lower ratio of similar vs. dissimilar alignment with mouse cells when analyzing human healthy cells (Supplementary Figure S17d) rather than human diseased cells (Supplementary Figure S17e). A possible explanation for that may be the fact that not all mouse disease model fibroblasts actually have an inflammatory phenotype [60], hence some of them may be mixed with human healthy fibroblasts (Supplementary Figure S17d**)**.

However, in an unbalanced skin dataset setting, GLUE no longer outperformed other methods. To create an unbalanced dataset, we removed human disease samples from the integration. This presented an additional challenge for both VAMP + CYC and GLUE, leading to potential over-integration of the diseased mouse and the healthy human cells. It is thus important to keep in mind that no integration is perfect [26] and thus the conclusions drawn from the integrated embedding should be generally seen as a source of new hypotheses rather than a ground truth.

Despite these challenges, VAMP + CYC presents a more robust choice since it was less strongly affected by this complex setting. The shift in diseased-healthy mixing between pre- and post-ablation settings was more substantial in GLUE than in VAMP + CYC (Supplementary Figure S17d, f). Strong discrepancies in conclusions obtained under different dataset designs are a big problem for real-world use cases, such as atlas-based analysis. For example, atlas may be evaluated on a subset of conditions with balanced data across systems and afterwards used for mapping a different query condition, which does not contain balanced data. Subsequent strong shifts in performance between the atlas, which was extensively evaluated, and the query mapping use-cases, which often rely on atlas quality, can thus lead to over-confidence in conclusions drawn about query data.

We also note that our automatic hyperparameter selection approach chose an unusually high cycle consistency loss weight (weight = 50) and subsequently batch correction strength for VAMP + CYC for the unbalanced dataset. This hyperparameter choice was substantially above choices for other datasets (Supplementary Figure S1, weight = 2 or 5) and what is recommended in our integration tutorial (weight around 5). If a more reasonable cycle consistency loss weight is considered, the increase of the healthy-diseased mixing in the pre- and post-ablation setting is less substantial (Supplementary Figure S18). This is not the case for GLUE, where all hyperparameter settings led to substantial shifts in pre- and post-ablation healthy-diseased mixing across species (Supplementary Figure S18). Thus, by removing the imperfections of our automatic hyperparameter selection approach and instead considering general method behaviour, the higher robustness of VAMP + CYC compared to GLUE against unbalanced data becomes more evident, as also observed in the retinal data.

We attempted to perform a similar cross-species analysis for healthy and type 2 diabetic beta cells in the pancreatic dataset. However, in this dataset, none of the methods could consistently sufficiently distinguish diseased cells of one species from healthy cells of the other (Supplementary Figure S17b, c). This may be explained by the fact that already human beta cells alone do not separate between healthy and diseased donors, which was highlighted as an issue by the authors of the original human dataset publication [62]. Moreover, it was before reported that mouse type 2 diabetic models do not perfectly mimic human disease [1]. Nevertheless, the beta cell biological variation was preserved at least within the mouse data alone when using VAMP + CYC, as described above (Fig. 6b).

Overall, our results show that VAMP + CYC and, in some cases, GLUE can integrate data sufficiently well to draw meaningful conclusions about cross-system similarity of biological conditions. Other methods were generally unable to correctly align cross-system biological conditions. However, GLUE should not be used for datasets with unbalanced conditions across systems (i.e. with certain conditions missing from some systems) as it leads to severe loss of true biological differences. Thus, as unbalanced data is common in practice, VAMP + CYC presents a more reliable choice for real-world applications. Furthermore, as we also observed strong loss of fine-grained biological information in GLUE, this further advocates against its use. Overall, VAMP + CYC presents the best choice across different downstream use-cases.

## Discussion and outlook

In order to improve the integration of scRNA-seq datasets, in particular in the presence of substantial batch effects such as different protocols, species, or in vitro vs. in vivo, we explored the shortcomings of commonly used cVAE-based approaches for increasing batch correction, namely, KL regularization strength and adversarial learning. Models relying on these approaches struggled with retaining sufficient biological information when increasing batch correction. We observed similar limitations in established integration methods that are not based on the cVAE architecture. To overcome these challenges, we proposed the use of the VampPrior and latent cycle-consistency loss. We performed evaluation in three data scenarios (cross-species, organoid-tissue, and cell-nuclei) and observed consistent model performance characteristics and failure modes, enabling us to make recommendations regarding individual models. We found that our model, which combines the VampPrior and cycle-consistency loss, had an overall good performance in both batch correction and biological preservation as well as enabled truthful interpretation of the integrated embedding, making it our method of choice. To make this model easily accessible to the community, we also implemented it in the scvi-tools package [42] under the name sysVI.

Our proposed method is both computationally efficient and scalable, making it well-suited for analyzing ever-growing single-cell datasets. It demonstrates linear scalability with respect to the number of cells (Supplementary Figure S19a), as shown by its successful application to the two-million-cell retina atlas. Although its runtime is approximately double that of scVI (Supplementary Figure S19b), a difference attributed to the computational costs of the VampPrior and cycle-consistency loss, its overall efficiency remains highly competitive. For example, it can process half a million cells in under 50 min (Supplementary Figure S19a), whereas methods like Seurat can require over 20 h for similar large datasets. Furthermore, its memory requirements are only marginally higher than scVI’s (Supplementary Figure S19c, d). This combination of robust integration performance, scalability, and manageable resource usage makes sysVI a practical and robust tool for large-scale data integration, particularly in the presence of substantial batch effects.

Based on the findings presented in this work, there are multiple directions of cross-system integration that could be further explored. The VampPrior and cycle-consistency loss could be easily added to other cVAE-based integration tools. Therefore, we urge the method-development community to switch from using batch distribution-matching techniques, such as adversarial learning, to cycle-consistency-based approaches and to replace the standard Gaussian prior with the VampPrior. Combining these approaches with other cVAE extensions may contribute towards achieving the goal of holistic whole-organism and cross-species atlases. The flexibility of the VampPrior holds promise for representation learning on complex datasets with diverse cell types and the cycle-consistency loss further improves the removal of substantial batch effects present in such datasets. To achieve population-wide integration, these two approaches could be combined with scPoli [63], which enables the integration of a large number of batches via learning individual batch embeddings rather than relying on the commonly used one-hot encoding. As a step towards this goal, batch embedding is available in sysVI. Furthermore, we here focus on integration strategies that do not require any prior cell type annotation, as this information is often unavailable and can introduce biases. Nevertheless, supervised label-aware integration methods such as scANVI [64] may outperform non-supervised models [20]. Thus, future work could explore the combination of different prior-knowledge-based strategies with the unsupervised techniques proposed here. Additionally, while we here did not observe a benefit in more complex orthologue mapping for cross-species integration, this may be of greater importance when integrating more evolutionary divergent species. Thus, future work could explore the effect of different gene mapping strategies, both as part of data preprocessing [19] as well as in the model internally, such as by enabling flexible gene relationships [5, 34] or using gene embeddings [53, 65].

One unexpected finding of this work was that the VampPrior led not only to improved biological preservation, as would be expected, but also to increased batch correction. A similar effect was also observed when using a simpler GM prior. However, the VampPrior showed overall better performance and higher robustness to varying numbers of prior components. Further work will be needed to fully understand the mechanisms of this phenomenon. These results may not only have applications for scRNA-seq integration but also in other domains using cVAE models for covariate effect removal.

While we have shown that VAMP + CYC model enables good cross-system integration in comparison to existing methods, it is not obvious which combination of data analysis decisions will lead to optimal performance and whether integration is the ideal approach at all. For example, here we have focused on evaluating different model architectures but did not analyze alternative data preprocessing decisions that may affect the final result, such as the approach used for selecting features across systems. While previous work assessed some preprocessing options [19, 20], it is unclear if the findings translate to all cross-system data use cases, which can make different assumptions about relationships between systems and their features. Moreover, while our results show that the optimal hyperparameter ranges are relatively similar across data use cases, the preferred setting will depend on the downstream application at hand. For example, annotation transfer across systems would benefit from stronger batch correction, while comparative analysis of systems may require preserving more biological differences in order to assess their true similarity. Furthermore, the coarse biological preservation metric we used (NMI) relies on matching cell type annotations across systems. However, as shown above for the retinal pigment epithelial and Mueller cells, sometimes cell types with the same name in different systems are, in fact, biologically distinct and should thus not be aligned, leading to biases in metric interpretation. Instead, one could use simulated data for evaluation. However, as simulations often cannot fully encompass the complexity of the real data they are likewise not an optimal solution [66]. Therefore, the community would highly benefit from standard benchmarking datasets where proper alignment is more carefully studied, reducing biases in integration evaluation.

While integration eases cross-system comparisons, there are also arguments against performing integration. For example, how strongly to integrate depends on downstream applications and is commonly assessed based on assumptions about the correct system alignment. Therefore, since the final integration is biased toward analysts’ expectations, it may not represent the biological ground truth. The integration also always removes some biological information. This is especially problematic if the integrated systems have substantial biological differences, as much of the biological variation would be lost to enable system alignment. One such example could be the integration of early-stage organoids with adult human tissue, as it is likely that the overlaps between biological functions of cell populations are minimal. In this case, per-system analysis followed by between-system comparison may be preferred. Lastly, recently emerging foundation models claim to obtain batch-free representations of cells via training on a large number of diverse datasets [67, 68], potentially removing the need for future data-use-case-specific integrations. However, as optimal integration strength often depends on the application, it is unlikely that one-size-fits-all models will be able to fully replace data-specific integration. Data-specific tuning of foundation models could improve performance, however, this is computationally expensive due to the large number of parameters. Therefore, classical integration models are likely to remain of importance in the foreseeable future.

In conclusion, we proposed an improved strategy for integrating datasets with substantial batch effects that combines a cVAE model with VampPrior and cycle consistency loss. This will ease comparative analyses across a wide spectrum of biological questions, thus better leveraging available scRNA-seq datasets.

## Methods

### Overview of cVAE-based integration approaches

In this study we focused on cVAE-based integration approaches and their extensions that improve integration by modifying the VAE objective (Fig. 1d). Here we provide a brief description of these methods.

In cVAE models, the encoder ($\:{E}_{\varphi\:}$) embeds cell expression ($\:x$) and batch information ($\:c$, which also contains system information), into a batch-effect corrected latent representation ($\:z$). Then, a decoder ($\:{G}_{\theta\:}$) reconstructs the expression based on latent representation and batch information. Two opposing losses are used to train the model (combined as $\:{L}_{cVAE(\theta,\phi)}$), the expression reconstruction loss that promotes information preservation during encoding and decoding and $\:KL$ loss-based regularization of latent space that encourages information compression towards a Gaussian prior distribution.

$$L_{cVAE(\theta, \phi)} = -\mathbb{E}_{z\sim q_{\phi}(z|x,c)}[\log p_{\theta}(x|z,c)] + D_{KL}(q_{\phi}(z|x,c)||p_{\theta}(z))$$

#### Tuning of KL regularization strength in cVAEs

The most straightforward approach for increasing batch correction strength is using a higher $\:KL$ regularization loss weight. This leads to lower preservation of information within the latent space, as it pushes samples’ latent representations towards the Gaussian prior distribution. The information is removed not only for technical or batch variation, which is desired, but as a side effect also for biological variation [30]. To enable good initialization of biological representation the KL regularization loss weight can be gradually increased during training via annealing, as done in scvi-tools [42].

#### Adversarial loss

Multiple approaches have been proposed for promoting indistinguishability of latent distributions across batches, such as maximum mean discrepancy [31, 69], contrastive mixture of posteriors misalignment penalty [32], and disentanglement of batch and biology-related latent components [33]. We chose adversarial learning as an example due to its popularity in the single-cell community [34–36]. The adversarial classification loss ($\:{L}_{ADV}$) can be added to the $\:{L}_{cVAE}$ objective to promote indistinguishability of latent spaces of different batches [34–36]. For this, a discriminator network ($\:{D}_{\psi\:}$) is added, which is trained by minimizing classification loss ($\:{L}_{ADV}\:$) responsible for predicting the batch from the latent vector of each cell. In contrast, the encoder of the cVAE is trained by maximizing $\:{L}_{ADV}\:$, opposing the discriminator’s ability to distinguish between batches.

$$L_{ADV(\phi,\psi)}=E_{z\sim q_{\phi}(z|x,c)}[CrossEntropy(D_{\psi}(z),c)]$$

#### Cycle-consistency loss

Latent space cycle-consistency is an alternative to adversarial learning that works by pushing together the latent representation of matched cells from two batches. Cell $\:{x}_{i}\:$belonging to the batch $\:i$ is encoded into a latent representation $\:{z}_{i}$. That latent representation is then decoded with batch covariate $\:j$ into the cell $\:x{{\prime\:}}_{j}$, which represents the cell $\:{x}_{i}$ as if it originated from the batch $\:j$. The cell $\:x{{\prime\:}}_{j}$ is encoded into the latent representation $\:z{{\prime\:}}_{j}.$ Finally, the model penalizes the distance between the original representation $\:{z}_{i}$ and the cycle-generated $\:z{{\prime\:}}_{j}$ via an additional loss component ($\:{L}_{CYC}$). Here, different distance minimization losses can be applied, with our choice being the mean squared error (MSE) on standardized data.

$$z_{i} = E_{\phi}(x_{i},c_{i}) \\ z'_{j} = E_{\phi}(G_{\theta}(z_{i},c_{j}), c_{j}) \\ L_{CYC(\theta,\psi)}=E_{z\sim q_{\phi}(z|x,c)}[Distance(z_{i},z'_{j})]$$

#### The VampPrior

The VampPrior replaces the unimodal Gaussian prior $\:{p}_{\theta}\left(z\right)$ with a mixture of trainable Gaussian prior components (with the number of components $\:L$), giving it more representation flexibility. Their prior parameters are not defined in the latent space, as is usually done in cVAEs, but rather in the input cell space as “pseudoinputs” ($\:{xpi}_{l}$), which are passed through the encoder to obtain latent representations (mean, variance) used to parametrize the Gaussian components of the prior distribution. This thus directly couples the prior and the posterior. Additionally, the weights ($\:w$) of the prior components are likewise learned [40, 70].

$$p_{\theta}(z) = \sum_{l=1}^L Softmax(w_{l})q_{\phi}(z|xpi_{l})$$

#### Cell type supervision

Previous work has proposed using supervised training with prior cell type labels to improve data integration. This is typically achieved through two main strategies: contrastive training, where cells from the same cell type are pushed together and cells from different cell types may be pushed apart [53, 63], and classification loss, which ensures that latent space enables good cell type classification performance [64]. scANVI is an example of methods using cell type classification to improve integration [64]. Additionally, other approaches, such as constraining the cVAE’s prior distribution with cell type information, have been explored [71]. While in some cases cell type labels can be replaced by unsupervised cell clusters [53], the effectiveness of supervised learning is heavily dependent on the quality of this prior information. When this is not the case, serious integration mistakes may occur, as described above for SATURN. Since new data often lacks reliable annotations, supervised approaches are not ideal for general use. For this reason, we excluded supervised methods from our analysis and instead focused on the performance of unsupervised techniques.

### Data preprocessing

Five datasets for the three types of use cases were prepared separately as described below. For the mouse-human data, we prepared a pancreatic data composed of pancreatic islet datasets of mouse [1] (without embryonic and low-quality cells) and human [62], and a skin data composed of adult human skin [61] (without erythrocytes) and skin cells from a mouse model [60]. For mouse-human skin dataset we introduced an additional biologically unbalanced setup referred to as “limited skin mouse-human” where human psoriasis samples are removed. For the organoid-tissue scenario, we used a retinal dataset [7]. For the cell-nuclei scenari, o we used adipose dataset [72] (using the SAT fat type), and the human retina atlas [73]. We obtained published count data and cell annotation for all datasets (see *Data availability* section) and removed unannotated cells. Where necessary, we manually curated cell population names to match across studies used within individual integration settings. In each system, we kept genes expressed in more than 20 cells, and in the mouse-human OTO setting we also only kept OTO genes, while in the FO setting, we removed genes without unique gene symbols (required for SATURN). Data was normalized with Scanpy *normalize_total* and log-transformed, and 3000 HVGs (4000 HVGs for organoid-tissue) were selected per system, keeping the intersection of HVGs across systems, similarly as proposed before [20]. For GLUE, we computed a non-integrated principal component analysis (PCA) per system on scaled data using 15 principal components (PCs), and for SATURN, we used this data to compute prior clusters per system.

Non-integrated embeddings were computed on the same cells as used for integration evaluation (described below). The normalized expression prepared for integration was standardized per gene, followed by computing 15 PCs, neighbors, and UMAP.

### Evaluation of batch effect strength in unintegrated data

Batch effect strength comparison within and between systems within a data set case was performed by computing Euclidean distances between mean embeddings of cell type and sample groups in the PC space (15 PCs on scaled OTO data). We used only groups with at least 50 cells and cell types where both systems had at least three remaining samples. The significance of differences in distance distributions within and between systems was computed per cell type with the one-sided Mann–Whitney U test.

Batch effect strength analysis of the three data types was performed using average silhouette width (ASW) with systems as the batch covariates for individual cell types of every data use case, using the non-integrated embeddings. We adapted scIB metrics function [20] so that ASW scores were not reported as absolute values. We used ASW rather than iLISI metric as iLISI was not discriminative enough for the substantial pre-integration batch effects [74]. As the computation was performed on the PCA space with comparable dimension ranges across data use cases, the dimension range bias described in Supplementary Note S5 does not affect the comparison.

### Integration

We corrected sample and system-level batch effects during integration by adding them to the model inputs as one-hot encoded vectors. The $\:{L}_{CYC}$ and $\:{L}_{ADV}$ were computed only on the system covariate. We ran each model with any given hyperparameter setting, as described below, three times with different random seeds. To ensure a fair model comparison, we tuned hyperparameters aimed at regulating integration in all models and did not tune any other hyperparameters.

Our custom cVAE implementation was based on the scVI framework [42]. Unlike in scVI, we simplified the model by using the Gaussian log-likelihood on normalized log-transformed data for reconstruction loss and did not use KL weight annealing. We used the same number of layers, layer size, and dimensions as for scVI. To regulate batch correction strength we tuned the KL loss weight.

Additional extensions were added on top of our cVAE model. We implemented the VampPrior as described by the authors of the original publication [40] and did likewise for the GM prior, but with prior components representing points in the latent space. For the VampPrior, we initialized prior components by randomly sampling cells from the data, and for GM prior, we either used sampled data that we passed through the encoder before training or used random initialization with mean in the range [0,1) and variance of one. As in cVAE, we also tuned KL loss weight for the VAMP model. The cycle-consistency loss was computed between the latent representation of a cell ($\:{z}_{c=i}$) and its cycle-pair coming from the other system ($\:{z'}_{c=j}$) using MSE on data standardized within a minibatch separately for cells and their cycle pairs. In both CYC and VAMP+CYC models we tuned the cycle-consistency loss weight. For the ADV model, we adapted the MultiVI [75] adversarial classifier within the scvi-tools framework to discriminate between the systems. The weight of the $\:{L}_{ADV}$ (Kappa) is optimized.

All previously published models (scVI, GLUE, SATURN, Seurat, Harmony, Harmony-py, and scMGCA) were run with default parameters, except for the following changes. For scVI we used *n_layers* = 2, *n_hidden* = 256, *n_latent* = 15, *n_steps_kl_warmup* = 1600, and *gene_likelihood* = nb and we tuned *max_kl_weight* (KL loss weight). The parameters *n_layers*, *n_hidden*, and *n_latent* were modified according to recent papers that used scVI or similar models for large-scale data integration [1, 28] and were aligned with the settings in our cVAE-based models. We used the nb *gene_likelihood* as this is likewise a common practice and part of some of the scvi-tools tutorials. We adjusted *n_steps_kl_warmup* to ensure that the warmup ran correctly with the specific number of epochs required for our datasets. In GLUE, we tuned *rel_gene_weight* (gene graph weights), *lam_graph* (graph loss weight), and *lam_align* (alignment loss weight). In scMGCA, we tuned the W_a, controlling the weight of the adjacency matrix reconstruction in the loss. In SATURN, we used the provided ESM2 protein embeddings and tuned *pe_sim_penalty* (protein similarity loss weight). The number of epochs was set to a fixed value per method and dataset, depending on the number of cells. In Seurat, we tuned k.anchor, which determines the number of neighbors used to construct anchors. This parameter controls the trade-off between sensitivity and specificity in anchor identification, affecting the integration quality. For Harmony (in R) and its Python implementation, we tuned the theta parameter. This parameter controls the strength of batch effect correction, with lower values preserving more biological variation and higher values enforcing stronger correction.

### Integration evaluation

We performed evaluations on at most 100,000 randomly selected cells per dataset to reduce the computational cost, except for Moran’s I, where cells were selected as described below. We computed neighbors on the latent embedding directly, except where we specified that the embedding dimensions were standardized prior to neighbors computation. This data was also used for UMAPs. The non-integrated embedding was computed with 15 PCs on scaled OTO data, using the same set of cells as for the integrated data.

We describe the rationale for metric selection in Supplementary Note S5. For LISI and ASW metrics, we used implementations from scib-metrics Python package (https://github.com/YosefLab/scib-metrics), and for NMI, we adapted their implementation to set a random seed. We computed the NMI-fixed and Jaccard index by first computing Leiden clusters at high resolution (*r* = 2) and then assigning a cell type label to each cluster based on the most common ground-truth label. This annotation was used for comparison to ground-truth labels. For Moran’s I, we compared Moran’s I values between non-integrated and integrated data as follows. For the pre-integration Moran’s I computation, we kept sample and cell type groups with at least 500 cells and excluded doublets. For each group, we removed genes expressed in less than 10% of the group’s cells, computed 15 PCs and neighbors per group, and used them to compute Moran’s I on all genes. We set a cutoff on Moran’s I per dataset to keep from around a dozen to around 150 genes per group for integration evaluation. On integrated data, we then repeated Moran’s I calculation on the same cell groups and the selected genes. The final score ($\:mi$) was defined as a ratio of post- ($\:m{i}_{post}$) and pre-integration ($\:m{i}_{pre}$) values averaged across genes ($\:G$), samples ($\:S$), and cell types ($\:CT$).

$$mi=\mbox{avg}_{CT}\left(\mbox{avg}_{S}\left(\mbox{avg}_{G}\left(\frac{mi_{post(G, S, CT)}}{mi_{pre(G, S, CT)}}\right)\right)\right)$$

We selected the best hyperparameter setting per model over all tested hyperparameters. For every model, we scaled individual metrics to [0,1] across all runs computed for hyperparameter optimization and then computed the biological preservation score as the average of Moran’s I and NMI and batch correction as iLISI alone. The overall score was computed as a weighted average of biological preservation (weight = 0.6) and batch correction (weight = 0.4), similar to a previous benchmark [20]. The best hyperparameter setting was selected based on the average overall score across runs and for every hyperparameter setting we selected a representative run for UMAP plots as the run with the median overall score. We observed that optimal hyperparameter ranges were similar across datasets (Supplementary Figure S1) and we further discuss considerations for hyperparameter tuning in Supplementary Note S6.

We compared the integration performance of all the benchmarked integration methods with their top-performing hyperparameter settings as described above. We used Welch’s t-test followed by multiple test correction per dataset and integration metric with the two-stage Benjamini and Hochberg method. However, a limitation of our analysis is that we always had only three samples per group due to the resource intensiveness of integration benchmarking, reducing the statistical power.

Preservation of gene groups known to be variable within pancreatic islet beta cells of healthy adult mice was evaluated with Moran’s I on the control sample from the mSTZ dataset [1]. We used Scanpy *score_genes* to obtain a single score for every gene group and computed Moran’s I on these scores for every embedding.

Biological conditions across systems were compared with iLISI metric. For each setup, we defined a pair of similar cell groups (e.g., healthy cells from both systems) and dissimilar cell groups (e.g., healthy cells from one system and diseased cells from the other systems) on a specific cell type. Then, we measured the mixing of cell groups by subsetting only to the cells from these groups and applying iLISI with system as a batch. We favor models with low iLISI for dissimilar groups and high iLISI for similar groups.

## Supplementary Information

Supplementary Material 1.

## Data Availability

The datasets were retrieved from public repositories. Pancreas islets - mouse: GEO (GSE211799), https://cellxgene.cziscience.com/collections/296237e2-393d-4e31-b590-b03f74ac5070. Pancreas islets - human: www.isletgenomics.org. Adipose: Single Cell Portal (SCP1376). Retina organoid and primary tissue: https://cellxgene.cziscience.com/collections/2f4c738f-e2f3-4553-9db2-0582a38ea4dc. Human retina atlas: https://cellxgene.cziscience.com/collections/4c6eaf5c-6d57-4c76-b1e9-60df8c655f1e. Skin - mouse: https://cellxgene.cziscience.com/e/7be2824e-d62a-42ef-9169-084555c6df87.cxg/. Skin - human: https://cellxgene.cziscience.com/e/b796f27e-e191-4f2d-b40c-e6ac58163b4e.cxg/. The model and analysis code as well as the conda environment specifications are available at: https://github.com/theislab/cross_system_integration. We implemented our method in scvi-tools package as an external model named sysVI (https://docs.scvi-tools.org/en/latest/api/reference/scvi.external.SysVI.html*)* and provided a tutorial at: https://docs.scvi-tools.org/en/latest/user_guide/models/sysvi.html.
