# Supplementary material for: Integrating single-cell RNA-seq datasets with substantial batch effects
Source: BMC Genomics. 2025 Oct 30;26:974. doi: 10.1186/s12864-025-12126-3 (PMC12577435; doi:10.1186/s12864-025-12126-3)
Supplement: Supplementary file 1 — Supplementary Material 1. [file 12864_2025_12126_MOESM1_ESM.pdf]

Supplementary materials

Supplementary notes

Supplementary Note S1: **KL regularization strength tuning leads to dimension shrinkage, but not batch correction**

Increasing KL regularization strength led, as expected, to stronger batch correction and lower biological preservation. However, we observed that this was not associated with the indistinguishability of cell representations in the latent space, but rather was caused by a reduced number of latent dimensions effectively used in downstream cell graph computation. Namely, stronger KL regularization leads to more latent dimensions having variation across cells near zero and thus cells having a value of individual latent dimension close to zero as the distributions are zero-centered (**Supplementary Figure S2**). This occurs as the cell embedding is the predicted mean of the cell's latent variable representation, as is also done in scVI and GLUE, which is pushed towards zero to match the Gaussian prior when increasing KL regularization. As neighbors graph computation is standardly done with Euclidean distance on non-scaled embedding this effectively leads to a small number of latent dimensions having a stronger effect on the graph representation. Indeed, after applying standard scaling to the embedding prior to the neighbor graph computation the different KL regularization strengths no longer strongly affected NMI and iLISI (**Figure 2a,b**, **Supplementary Figure S4**, **Supplementary Figure S3**). This indicates that biological and batch information is preserved in the embedding, but is not captured by the standard graph computation protocol. Nevertheless, we observed a drop of within-cell-type variation (Moran's I) on both scaled and unscaled data when increasing KL regularization strength, suggesting that finer variation is nevertheless lost. In contrast, while increasing L_{CYC} loss weight also led to the shrinkage of latent dimensions, the embedding was not sensitive to scaling. We achieved this by computing cycle distances on latent embedding standardized within a minibatch. Thus, while KL regularization strength tuning in standard cVAE merely leads to a lower number of used latent dimensions, thus reducing both biological and batch information at the same time, L_{CYC} actually removes batch effects from the embedding, as indicated by higher batch correction at comparable biological preservation (**Supplementary Figure S1**).

Supplementary Note S2: **Learning of prior components in VAMP**

The VampPrior, as proposed in the original publication[40], contains learnable components and during training the components evolve to be located within data-dense regions (**Supplementary Figure S13**). Interestingly, if prior components are fixed, this does not affect VAMP integration performance, but strongly affects GMM, where batch correction drops to a similar level as when using a single prior component (**Supplementary Figure S8**). This is somewhat unexpected, as one would assume that the encoder would be better able to align input representations to fixed prior components when inputs and prior parameters do not lie in the same space, as is the case in the GMM, but not the VAMP.

We also inspected the effect of prior component initialization. VAMP pseudoinputs were initialized with input data. We did not use random initialization as it is challenging to perform appropriate random initialization of pseudoinputs in the input space that would match the input cell distribution and could thus be effectively encoded by the encoder, which is trained to generate input cell representations. In the GMM, the data-based and random prior initialization showed no clear differences (**Figure 3**). Furthermore, the choice of input cells used to initialize pseudoinputs in VAMP did not seem to play a role, with similar performance when initializing them from a single cell type or system or in a balanced fashion from all cell types or systems (**Supplementary Figure S9**). This indicates robustness against varying prior initialization.

Supplementary Note S3: **Excessive reliance on prior cell cluster labels in SATURN harms cell representation learning**

SATURN uses prior cell cluster information to guide cell representation fine-tuning of a pre-trained conditional autoencoder (cAE)-based embedding. This is achieved by maximizing within-species distances between cells with different labels and minimizing cross-species distances between cells that are likely to have the same label. This leads to good cell-type level preservation when high-quality cell-type labels are available per species (SATURN-CT), however, it does not capture well within cell-type information (**Figure 5a**). This can be explained by the lack of cell

expression representation objective in the fine-tuning step, which relies on labels alone. Moreover, the contrastive loss directly pushes cells within cell types together, thus removing within-cluster variation. Thus, it is likely that even an implementation where contrastive loss is used during cVAE training, as in scPoli[63], may suffer from poor within-cell-type information preservation. In contrast, classification-based loss, for example, in scANVI[64], may be better suited for preserving within-cluster variation as it only enforces that cell clusters separate in latent space, but does not enforce any constraints on within-cluster structure. However, this hypothesis would need to be further tested.

Furthermore, the prior labels must be of good quality to lead to proper cell type separation. While the authors propose that clusters can be used as a source of prior label information, we observed that this led to poor NMI (**Figure 5a**). Namely, when multiple batches are present within species, as the datasets in our pancreatic mouse data, this will result in multiple dataset-specific clusters for individual cell types, which will also be reflected in the separation of cells by prior cluster in the final integration (**Supplementary Figure S14**). Similar effects were also observed when prior clusters were too fine or coarse with respect to underlying biological variation. For example, immune cells were separated into three distinct clusters when using cluster-based prior (SATURN), directly corresponding to prior clusters, and were merged into a single cluster when using cell-type-based prior (SATURN-CT). In both cases, the finer cell cluster structure retained within other models was lost (**Figure 5b**).

Supplementary Note S4: **Excessive reliance on batch covariate information leads to over-integration**

Downstream interpretation relies on striking the right balance between aligning cell populations that differ primarily due to batch effects and separating biologically distinct populations. For this, the relevant biological variation and undesired batch effects between systems must be disentangled. This is possible if the batch effects are more consistent across cell types, while system-specific biological variation, such as disease-induced heterogeneity, is cell-type-specific, which may often be the case. Thus, if dissimilar cell types are embedded in the same region of the latent space, this will lead to higher reconstruction loss as the batch covariate alone will not be able to explain this variation. However, if reconstruction quality is disregarded after the batch correction step, as described below for scGEN[59], this may lead to over-integration.

The scGEN model first trains a normal cVAE and then applies an additional batch correction step to every cell type separately. This is done by selecting one batch as the reference and moving all other batches (queries) on top of the reference batch. First, the distance between the reference and query mean latent embedding is computed and then query cell embeddings are transformed by the addition of the distance vector. This will force batch overlap even in the presence of biological differences, making this approach inappropriate for most data use cases as batch and biological effects are usually not orthogonal. For this reason, we did not include scGEN in our benchmark. Nevertheless, we show below that scGEN indeed fails to correctly align biological populations.

All models except scGEN correctly separated retinal pigment epithelial cells from organoid and primary tissue data[7] (**Supplementary Figure S5a**). In the Mueller cell example, where organoid cells are expected to align with the tissue periphery but not fovea cells[7], scGEN with samples as batch covariates led to over-integration, placing all samples on top of each other (**Supplementary Figure S11**). The alignment was also not improved by using systems rather than samples as the batch covariate, which instead led to under-integration on the sample level within systems. This also resulted in poor alignment of systems as the alignment with latent space arithmetic was disrupted due to the per-system embedding structure and its mean being strongly affected by sample batch composition rather than being dominated by biology (**Supplementary Figure S5a, Supplementary Figure S11**). This highlights another issue, namely that there is no guarantee that the alignment will be correct on the level of cell subtypes for which the corresponding cell annotation is unavailable. This is a consequence of the model never being faced with the task of reconstructing cell expression from the final corrected latent space. For example, we observed some errors in the amacrine subtype alignment, where cells expressing starburst amacrine marker *SLC18A3* did not co-localize in the scGEN embedding while showing clear sub-localization when using the VAMP+CYC model (**Supplementary Figure S15**). Therefore, we deem scGEN inappropriate for integration as its latent space is inherently biologically flawed due to over-reliance on prior information about cell types and batch covariates.

Supplementary Note S5: **Selection of integration metrics**

Recently, multiple metrics for the evaluation of integration methods were proposed and implemented in the scIB package[20]. However, we decided to use only a subset that we believe enables a higher-quality evaluation. Indeed, it was before reported that some metrics are biased and should thus be excluded from evaluation[21, 74]. Likewise, we observed that metrics that operate on distances directly (such as ASW) rather than on the graph (such as LISI and NMI/ARI) lead to biases when more or less latent dimensions have a high-value range. We observed that when tuning integration hyperparameters, which leads to shrinkage of some latent dimensions (**Supplementary Figure S2**), metrics such as ASW led to inaccurate results that are, in the most extreme cases, even opposite to graph-based metrics and visually distinct patterns on UMAPs. To validate this, we simulated data (**Supplementary Figure S12a**) where one dimension had a bimodal distribution to represent two distinct groups and 15 additional dimensions represented Gaussian noise unrelated to the two groups. As a starting point, all noise dimensions had the same standard deviation. We then progressively shrank some of the noise dimensions to have a smaller standard deviation, thus corresponding to dimension shrinkage observed when tuning integration hyperparameters. When increasing the number of shrunken noise dimensions, but keeping the group dimension constant, the ASW-based metrics were much more strongly affected than the LISI-based metrics (**Supplementary Figure S12b**). This can be explained by the graph structure being more robust to adding random noise than the underlying distances. As most downstream single-cell analyses are based on graph representations rather than on distances directly, we believe that graph metrics thus more realistically represent data characteristics relevant for analysis. From the group of graph-based batch correction metrics, we decided not to use graph connectivity as it has a poor detection limit[21] and kBET due to computational costs[21], and from biological preservation metrics we excluded ARI, as it is computed in a very similar manner as NMI, leading to predominately redundant results (see scIB reproducibility code[20]), and cLISI, as we observed a poor detection limit with the metric being at its maximum in most cases, as also reported before[20].

While the NMI metric assesses the presence of cell clusters, which are of importance for any downstream single-cell analyses, its interpretation is complicated due to different factors that lead to poor correspondence between embedding clusters and cell type labels. Namely, NMI will be low both when we have merging of multiple cell types, usually caused by too high batch correction, as well as separation of cell types into multiple clusters, which can be caused by different factors. For example, in SATURN, individual cell types separate into multiple clusters due to over-reliance on prior labels (**Supplementary Note S3**). In contrast, multiple clusters per cell type may also be a result of poor integration, leading to low NMI even when cell types separate well within batches. This is, for example, observed in GLUE with small alignment loss weight (0.005 or 0.0005, **Supplementary Figure S1**). Thus, in certain analyses where we wanted to strictly separate between cell type mixing, which is usually associated with excessive batch correction, and the ability to separate cells by cell types, indicating biological preservation within batches, we used an adapted NMI version (NMI-fixed). This metric uses a fixed clustering resolution and then annotates each cluster based on the majority ground-truth cell type. These annotations are used for NMI computation, thus indicating if cell types can be well separated. This corresponds to the ability to do standard scRNA-seq annotation, where clustering at high resolution followed by cluster merging is often used to annotate populations that require high resolution to form distinct clusters. However, NMI-fixed cannot detect over-clustering as observed in SATURN, thus we decided to use NMI in most other analyses as it is more generally able to detect if the integrated clusters directly correspond to cell types.

The previously suggested biological preservation metrics[20] are based mainly on cell type labels, which are commonly available only for cell types, but not for cell states within cell types. Some metrics, such as trajectory, cell cycle, or HVG conservation enable biological preservation evaluation at the sub-cell type level, however, they are often not applicable due to the lack of a trajectory or cycling cells in the data and integration methods not producing corrected expression values, respectively. Thus, to evaluate how biological variation is preserved within cell types, we adapted Moran's I biological preservation metric, which was proposed before[1] to measure spatial covariation of gene expression across an embedding, corresponding to distinct gene expression patterns. We changed the metric to first identify variable genes within individual non-integrated samples and cell type groups and then compare their variation before and after integration for every individual group. These metric modifications enable the selection of more relevant genes that are truly variable prior to integration, remove batch

effect biases due to per-sample computation, and enable direct comparison before and after integration. We show examples of Moran's I values and the corresponding expression patterns for five gene groups known to be variable in mouse healthy adult beta cells[1] on embeddings produced with different integration models (**Supplementary Figure S12c**). However, as this metric is computed per sample, it cannot detect the preservation of cross-sample patterns or proper alignment across samples. Sample alignment is much more challenging to measure as it usually requires prior knowledge about sample metadata, such as the presence of a developmental trajectory or disease-driven differences across samples within an individual cell type. Therefore, we evaluated sample alignment on a few biological use cases, which we also quantified with iLISI metrics. However, we do not propose a generic metric for it.

Supplementary Note S6: **Effect of hyperparameter tuning on the performance of different models**

One important characteristic of integration models is the ability to tune hyperparameters regulating batch correction to achieve sufficient integration for downstream analyses, depending on the batch strength. At the same time, increasing batch effect correction often reduces the preservation of biological information[20, 69], necessitating finding a good tradeoff. We observed that some models did not offer enough flexibility in batch correction tuning or contained an overwhelming number of possible parameter combinations to be accounted for. Furthermore, some hyperparameters weren't monotonically associated with batch correction, in contrast to our expectations.

In the scVI and VAMP models, stronger KL regularization resulted in lower biological preservation. However, iLISI did not increase consistently with increasing KL regularization strength and remained relatively low in all settings (**Supplementary Figure S1**, **Supplementary Figure S16**). This biological preservation-batch correction tradeoff makes this hyperparameter-model combination less favorable. In contrast, while increased L_{CYC} weight in CYC and VAMP+CYC models was also negatively correlated with biological preservation, this was somewhat less prominent than when tuning KL regularization strength, and the positive correlation with batch correction was also higher (**Supplementary Figure S1**, **Supplementary Figure S16**). Furthermore, L_{CYC} was also able to achieve higher iLISI than the tuning of KL regularization strength alone. Altogether, this makes L_{CYC}, in comparison to KL regularization strength, a better candidate for custom batch correction tuning of cVAEs, both when using a Gaussian prior and the VampPrior.

Multiple hyperparameters of GLUE (adversarial alignment loss weight, gene graph loss weight, and gene graph edge weight) affected integration performance in different ways (**Supplementary Figure S1**). We observed that increased alignment loss weight, which should increase adversarial batch correction, had an optimal iLISI range (around 0.05 in all datasets) and thus did not increase batch correction beyond a certain point, similarly as described for KL regularization in the scVI and VAMP models. Note that the low NMI at low alignment loss weight is not caused by over-integration but rather by the separation of cells across the system, thus leading to multiple clusters per cell type, which then do not match up with the cell type labels (**Supplementary Note S5**). Furthermore, decreasing gene graph loss weight and gene graph weights led to higher iLISI, with low gene graph weights leading to the highest iLISI overall, at the expense of biological preservation (**Supplementary Figure S1**, **Supplementary Figure S16**). This could be explained by the role of the gene graph in biological supervision, as it connects the gene embeddings and subsequently also cell embeddings from individual encoders. If the gene graph constraints are reduced, the gene correspondence between systems can be re-interpreted to increase batch correction. While this may be beneficial in moderate amounts, for example for cross-species integration, it could potentially, in extreme cases, lead to biologically false correspondence between genes and consequently cells. Furthermore, weaker graph constraints may lead to lower graph loss, with lower graph loss resulting in relatively higher importance of the alignment loss during training. Overall, the multitude of losses and their interactions in GLUE that affect integration performance complicates the selection of the hyperparameter set to tune.

As SATURN didn't offer many loss parameters to be optimized, we tuned only protein similarity loss weight that regulates the reliance on prior-defined protein embedding of genes. Despite a wide range of the tested values (0.01-10.0), we did not observe major variations in biological preservation or batch correction.

In the scMGCA model, we tuned the hyperparameter W_a, which adjusts the weight of the adjacency matrix reconstruction loss, testing values of 0.1, 0.3, 0.6, and 0.9. Lower W_a values (e.g., 0.1 and 0.3) yielded stronger preservation of biological information with no meaningful change in the batch correction capabilities.

Similarly, Harmony-py did not respond strongly to tuning the theta parameter. However, the R implementation strongly responded to changes in theta. The changes in both batch correction and cell type preservation were non-linear with an overall optimum at values around 5 or 10.

For Seurat, different k.anchor values were selected as optimal across datasets, clearly indicating the importance of fine-tuning this parameter. While the cell type preservation was negatively correlated with k.anchor values, the fine biological preservation did not change much across k.anchor values. The relationship between batch correction and k.anchor values differed across datasets.

It is important to consider that the observed hyperparameter-batch correction patterns hold only in the tested hyperparameter ranges. Nevertheless, the tested hyperparameter ranges were selected so that values outside of them would likely lead to inadequate biological preservation or batch correction, making them unsuitable for integration.

Supplementary methods

Simulation for comparison of graph and distance-based metrics

We simulated two groups of points (n=100 per group) from normal distributions with means of zero and one, respectively, and variance of one. We added 15 additional dimensions coming from a standard normal distribution, representing noise dimensions that do not separate between the two groups. ASW and LISI (both biological preservation and batch correction variants) were computed on all 16 dimensions while the size of 0 to 15 noise dimensions was divided by ten to push them towards zero. The simulations were repeated ten times.

Integration with scGEN

We ran scGEN with default parameters, except for *kl_weight=0.1*. We used either samples or systems as the batch variable since the model does not allow for multiple batch variables.

Supplementary figures

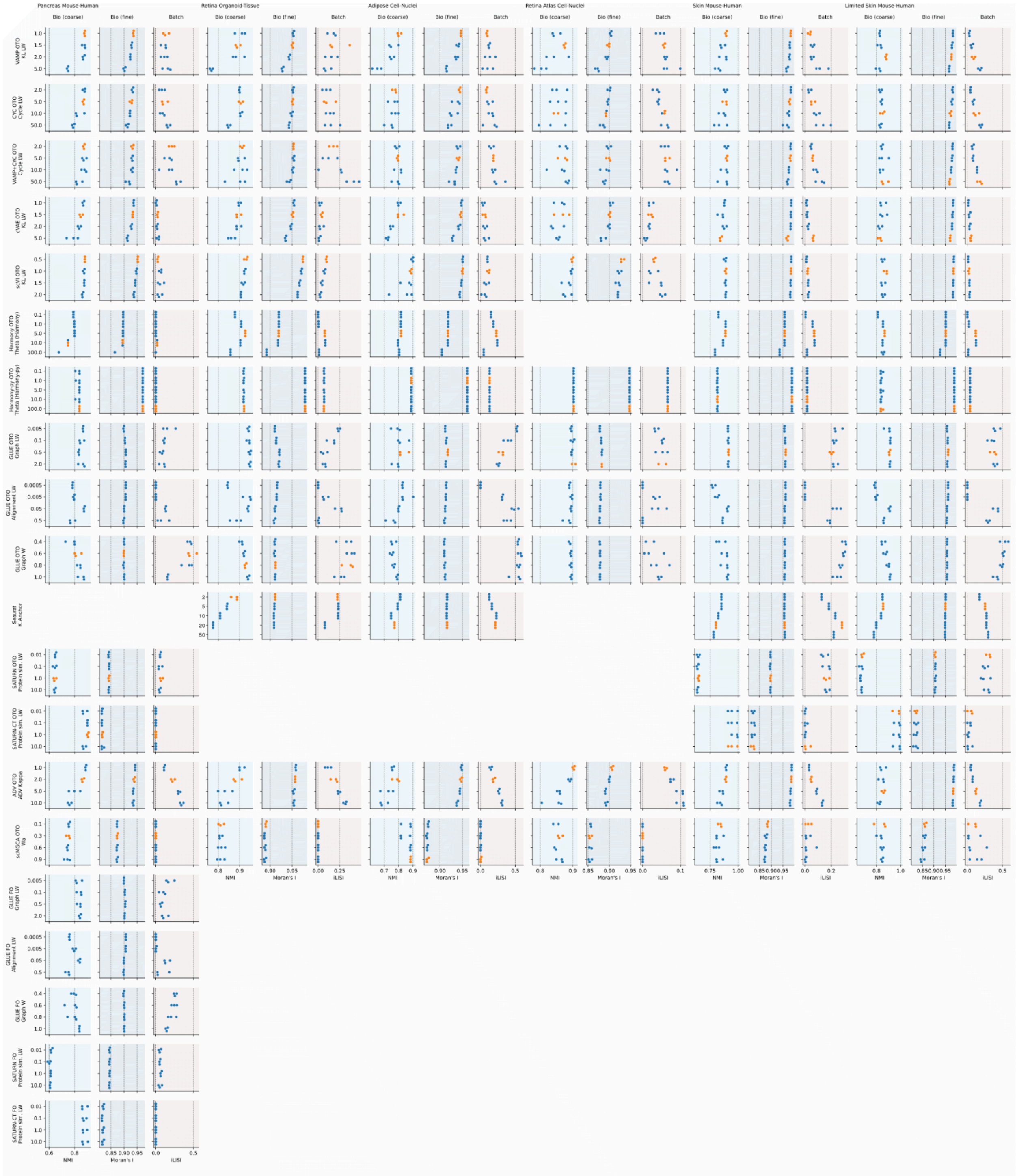

Supplementary Figure S1: Integration performance across models and different model hyperparameters. Rows show different models and tested hyperparameters (y-axis values), columns show per-dataset integration metrics, and dots represent individual runs with different seeds. For each setting, top runs are marked with orange. Limited skin dataset is the ablated version of the skin dataset used for the fibroblast use case.

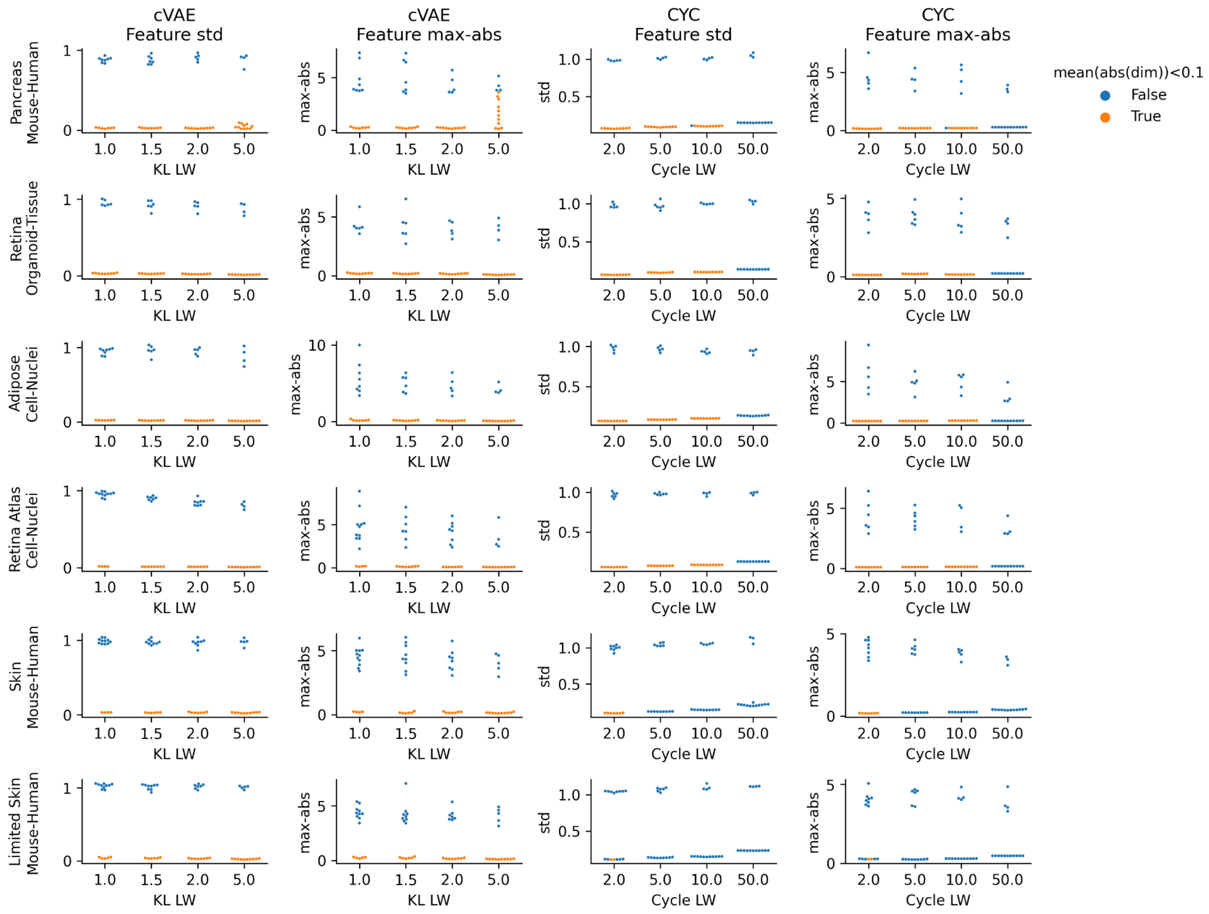

Supplementary Figure S2: Shrinkage of latent embedding dimensions when increasing batch correction-related hyperparameters. Standard deviations (std) and maximum absolute value (max-abs) of individual embedding dimensions (dots) obtained with different hyperparameter values (x-axis, shown for runs with seed=1) for cVAE and as a comparison CYC (columns) across datasets (rows). Each dot is colored orange if the mean absolute value in the corresponding embedding dimension is less than 0.1 and blue otherwise.

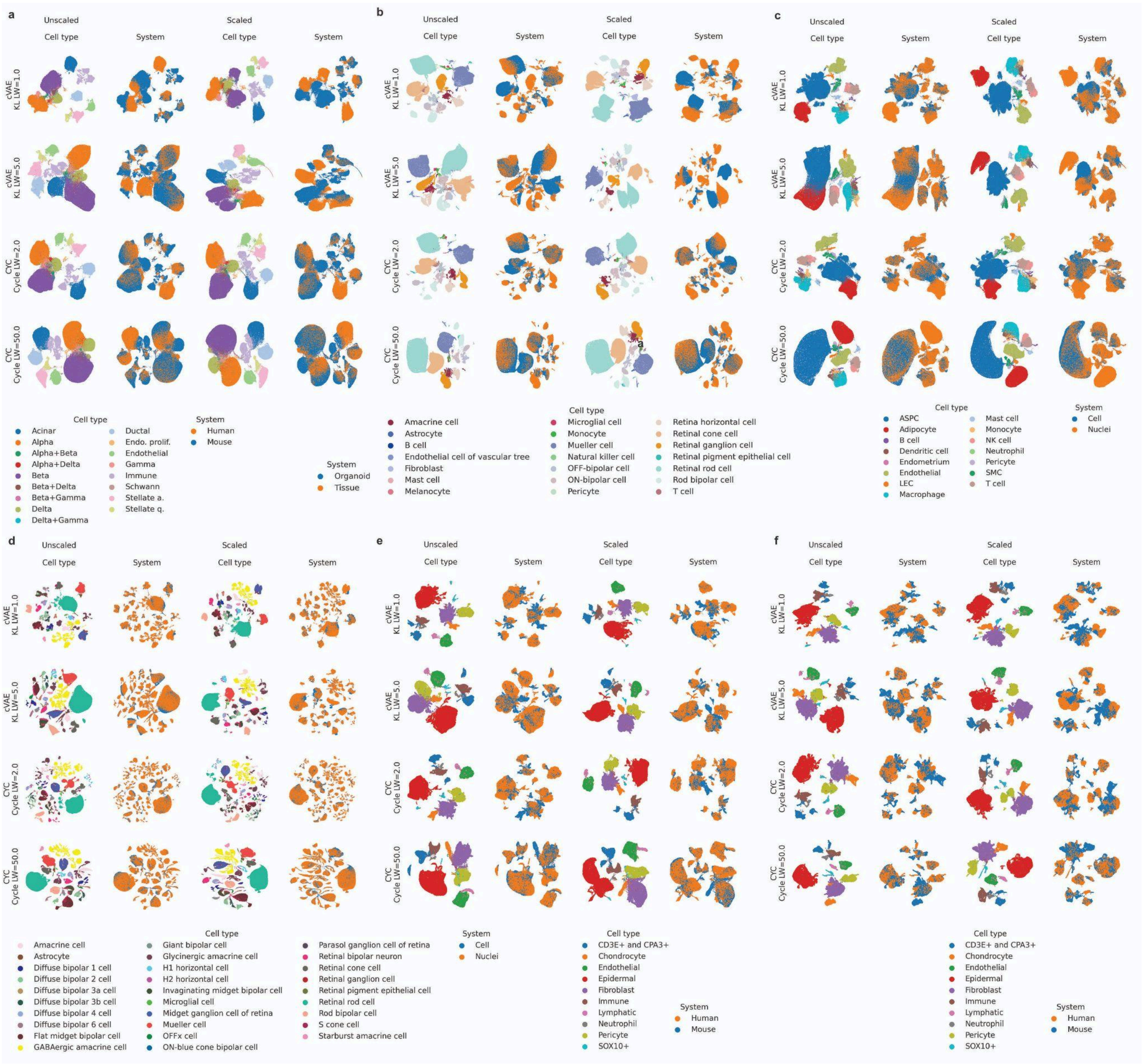

Supplementary Figure S3: UMAP of scaled and unscaled embeddings. Embeddings produced with cVAE and CYC models with low and high batch correction strength hyperparameter values (shown runs with seed=1). Datasets: (**a**) pancreas mouse-human, (**b**) retina organoid-tissue, (**c**) adipose cell-nuclei, (**d**) retina atlas cell-nuclei, (**e**) skin mouse-human, and (**f**) limited version of skin mouse-human.

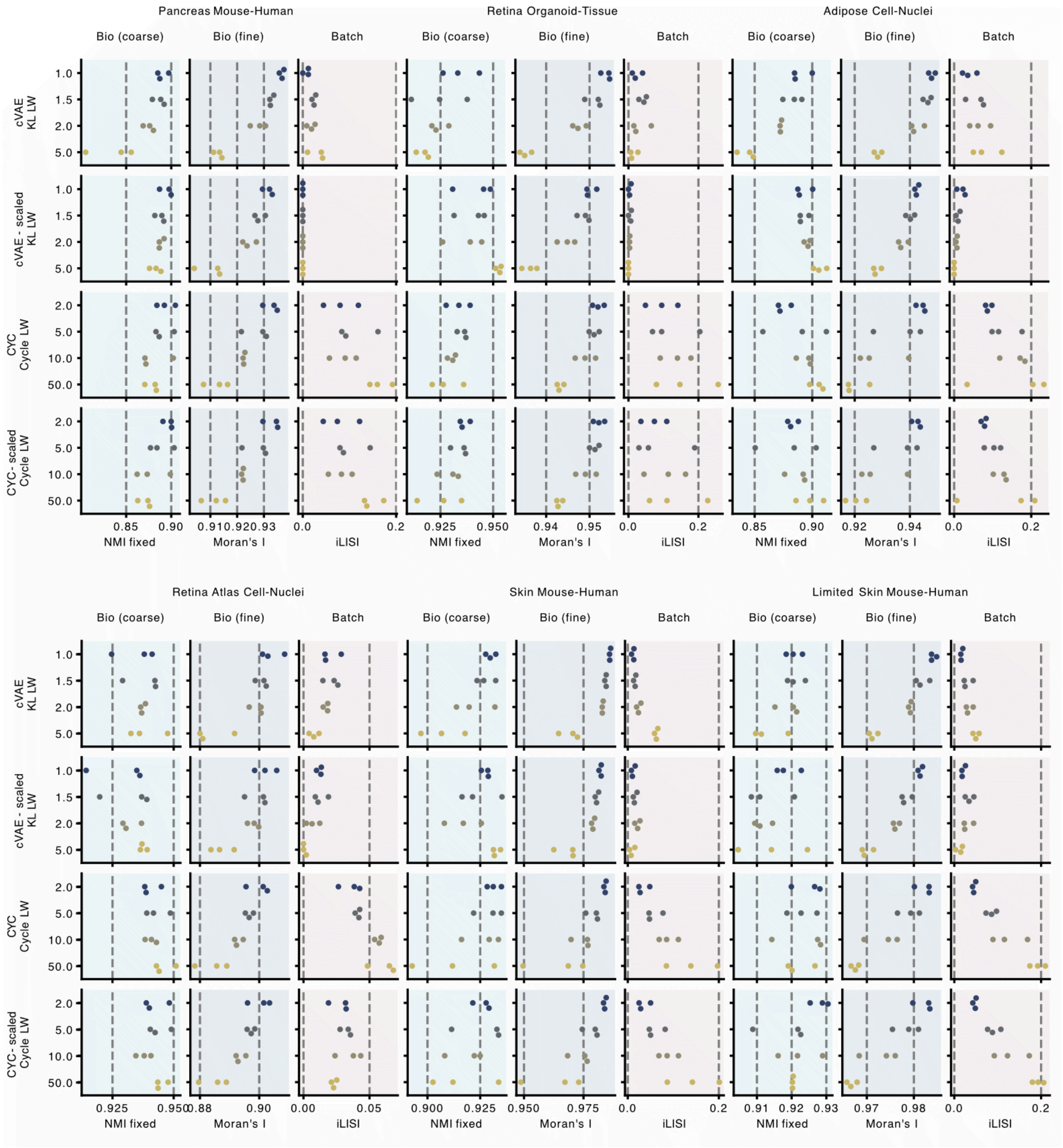

Supplementary Figure S4: Effect of scaling cVAE integration embedding on integration metrics. Integration metrics of datasets (x-axis) computed using scaled and unscaled embedding from cVAE and for comparison CYC, shown across different values of hyperparameters used for tuning batch correction (y-axis).

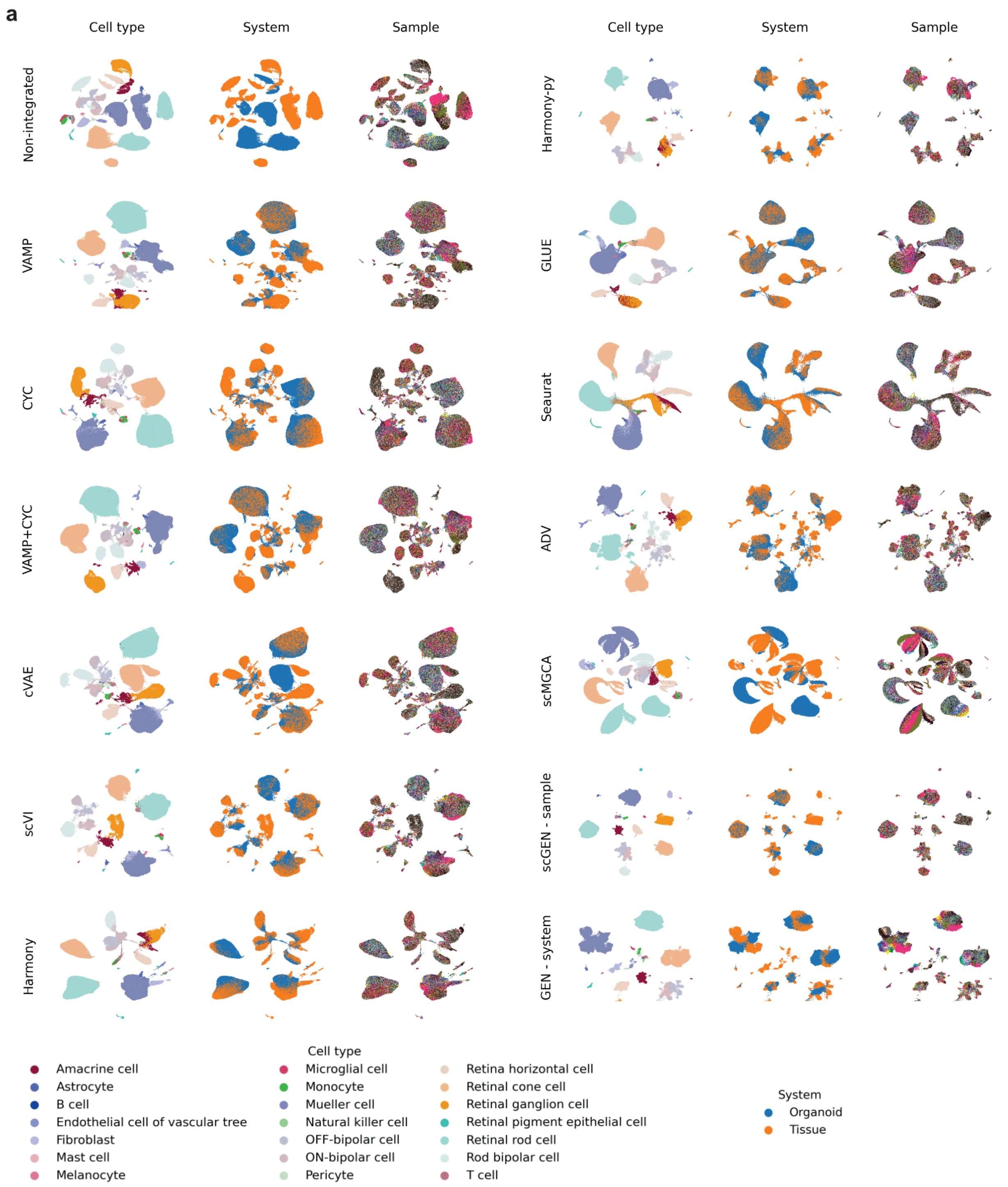
a
Cell type
System
Sample
Non-integrated
VAMP
CYC
VAMP+CYC
cVAE
scVI
Harmony
Harmony-py
GLUE
Seaurat
ADV
scMGCA
scGEN - sample
GEN - system
Cell type
Amacrine cell
Astrocyte
B cell
Endothelial cell of vascular tree
Fibroblast
Mast cell
Melanocyte
Microglial cell
Monocyte
Mueller cell
Natural killer cell
OFF-bipolar cell
ON-bipolar cell
Pericyte
Retina horizontal cell
Retinal cone cell
Retinal ganglion cell
Retinal pigment epithelial cell
Retinal rod cell
Rod bipolar cell
T cell
System
Organoid
Tissue

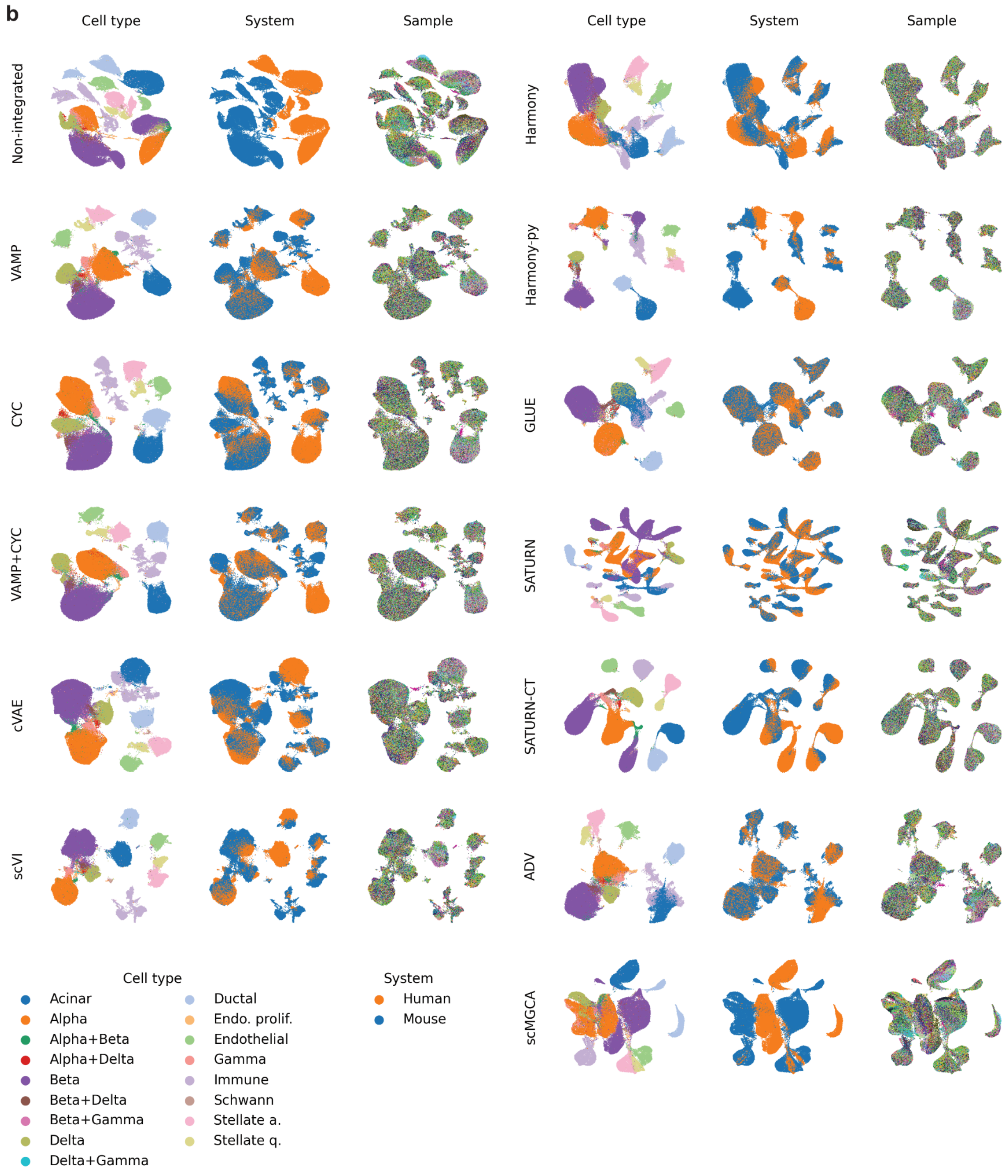

b
Cell type
System
Sample
Cell type
System
Sample
Non-integrated
VAMP
CYC
VAMP+CYC
cVAE
scVI
Harmony
Harmony-py
GLUE
SATURN
SATURN-CT
ADV
scMGCA
Cell type
Acinar
Alpha
Alpha+Beta
Alpha+Delta
Beta
Beta+Delta
Beta+Gamma
Delta
Delta+Gamma
Ductal
Endo. prolif.
Endothelial
Gamma
Immune
Schwann
Stellate a.
Stellate q.
System
Human
Mouse

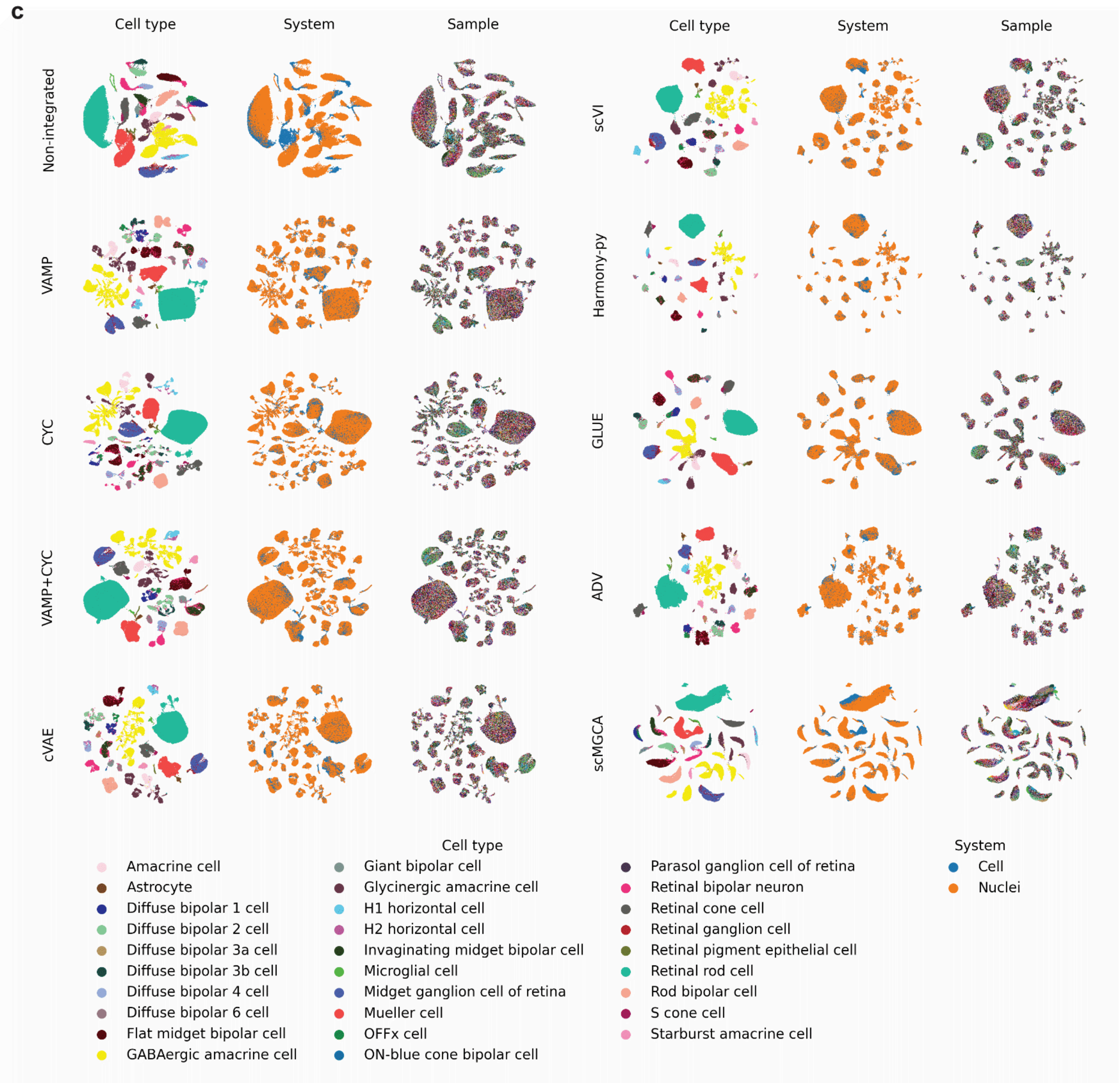
c
Cell type
System
Sample
Cell type
System
Sample
Non-integrated
VAMP
CYC
VAMP+CYC
cVAE
scVI
Harmony-py
GLUE
ADV
scMGCA
Cell type
Amacrine cell
Astrocyte
Diffuse bipolar 1 cell
Diffuse bipolar 2 cell
Diffuse bipolar 3a cell
Diffuse bipolar 3b cell
Diffuse bipolar 4 cell
Diffuse bipolar 6 cell
Flat midget bipolar cell
GABAergic amacrine cell
Giant bipolar cell
Glycinergic amacrine cell
H1 horizontal cell
H2 horizontal cell
Invaginating midget bipolar cell
Microglial cell
Midget ganglion cell of retina
Mueller cell
OFFx cell
ON-blue cone bipolar cell
Parasol ganglion cell of retina
Retinal bipolar neuron
Retinal cone cell
Retinal ganglion cell
Retinal pigment epithelial cell
Retinal rod cell
Rod bipolar cell
S cone cell
Starburst amacrine cell
System
Cell
Nuclei

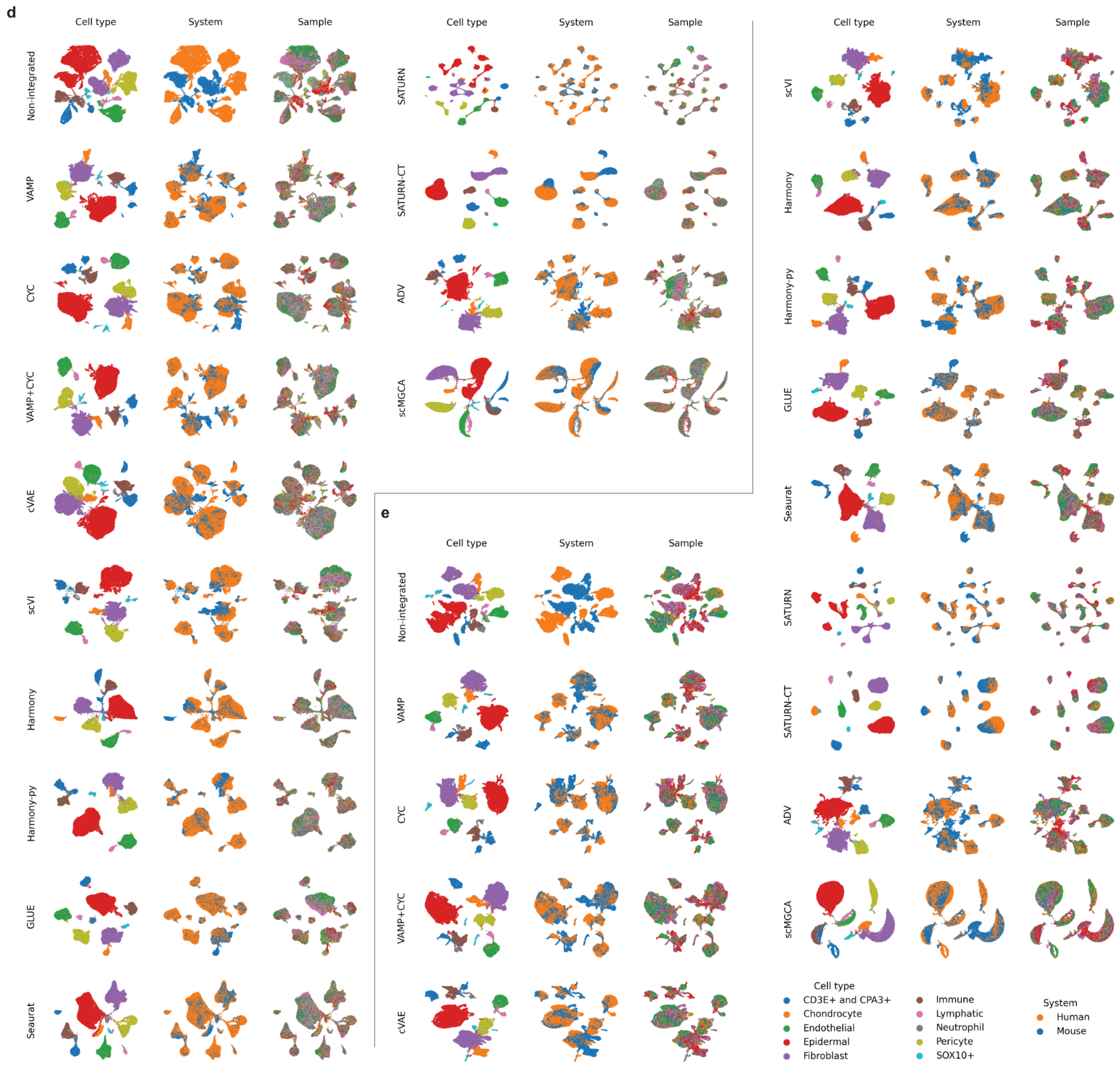

Supplementary Figure S5: UMAP embeddings of representative runs for the best hyperparameter setting of every model. Shown for datasets (**a**) retina organoid-tissue, (**b**) adipose cell-nuclei, (**c**) retina atlas cell-nuclei, (**d**) skin mouse-human, and (**e**) limited version of skin mouse-human dataset used for the fibroblast use case. To (**a**) we also added two example runs for scGEN with samples or systems as batch covariates. A legend for sample colors is not shown due to the large number of samples.

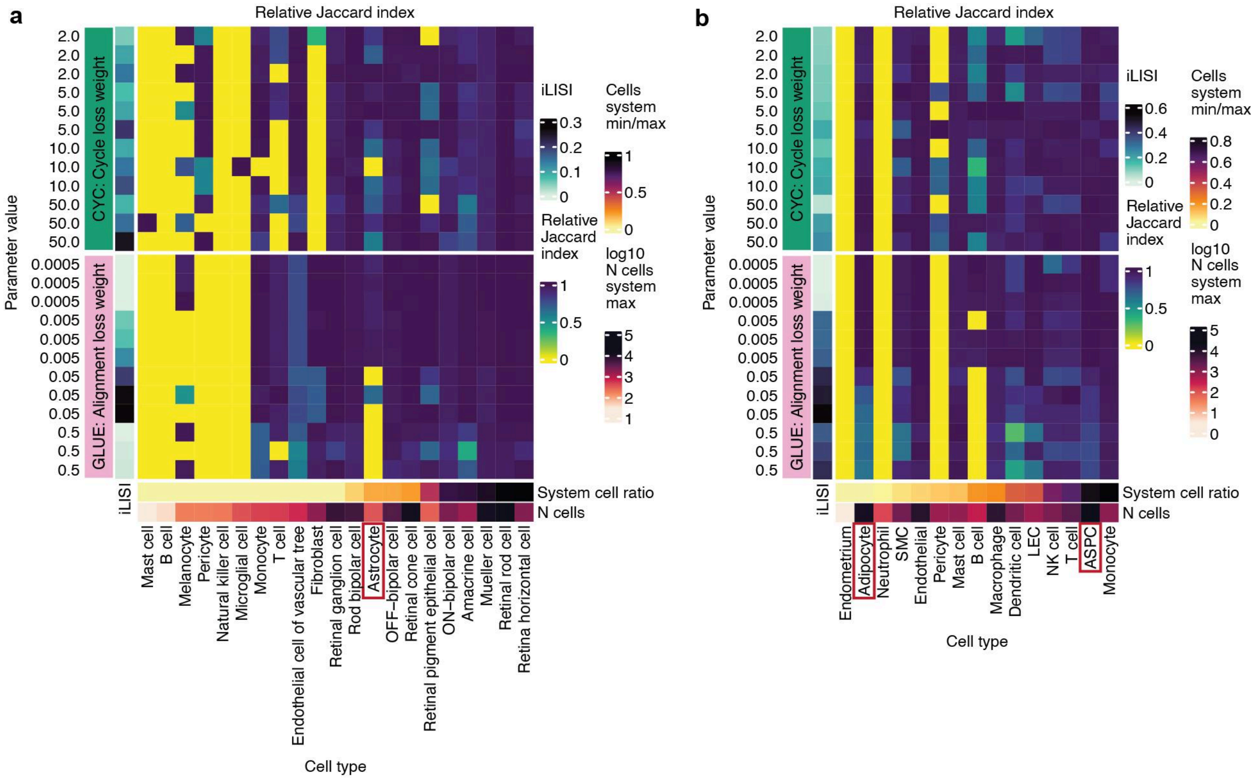

Supplementary Figure S6: Adversarial learning is more prone to cell type mixing than cycle-consistency loss. Shown is the cell type mixing score measured with the Jaccard index between embedding clusters and ground-truth labels; the score was max-scaled per cell type. Results are presented for integration of the (**a**) retinal organoid-tissue and (**b**) adipose cell-nuclei datasets with GLUE and CYC with different loss weights (LW) of losses that regulate batch correction. Individual cell types are annotated with the number of cells in the more abundant system and the ratio of cells between the less and the more abundant system. Red boxes mark example cell types that are commonly mixed by the adversarial model, especially when increasing batch correction strength.

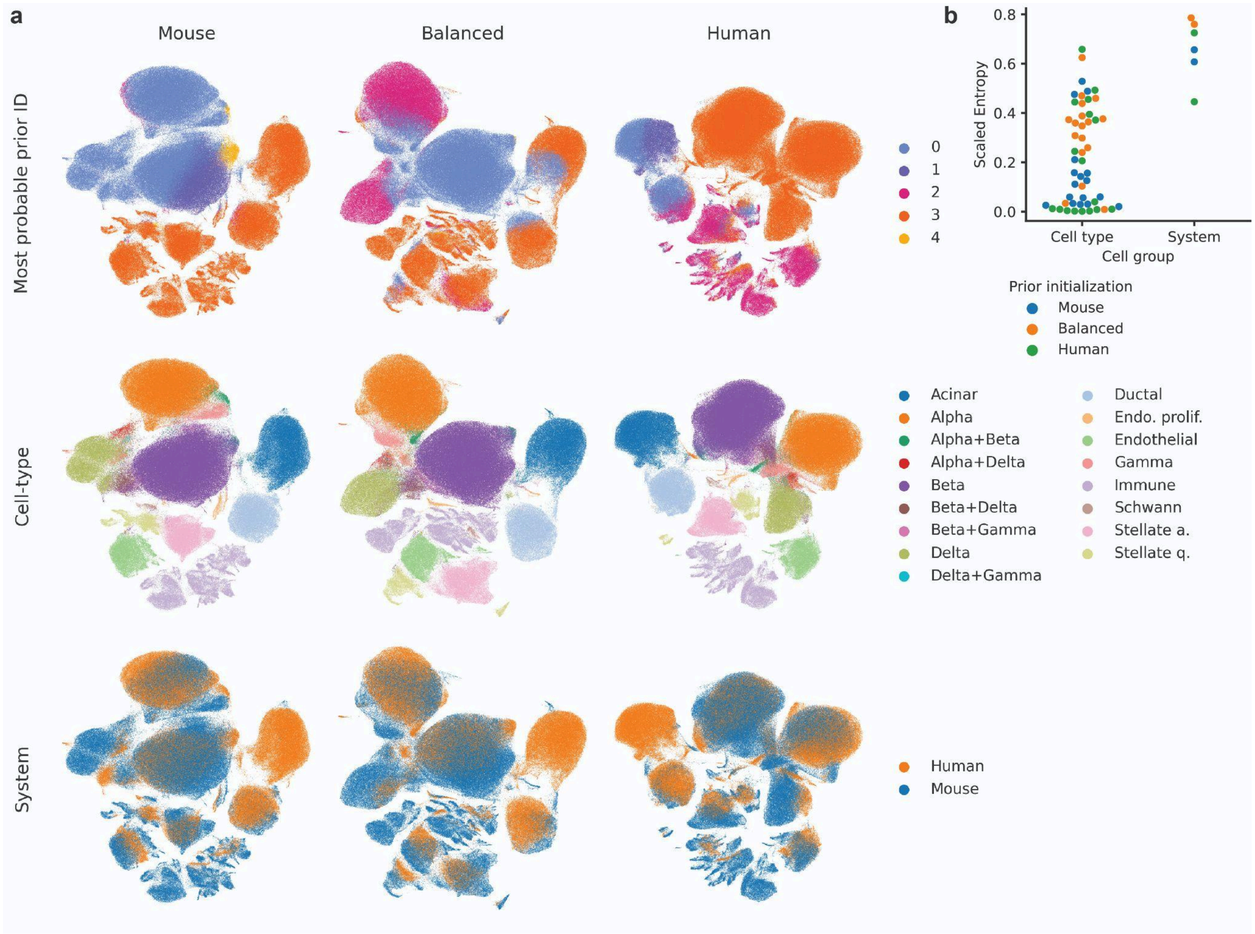

Supplementary Figure S7: Individual VampPrior prior components co-localize with cell types rather than systems. (**a**) Cells are colored by the prior component (N=5) with the highest probability, cell type, and system (rows) on embeddings generated with different prior initializations (columns). Priors were initialized either by sampling cells from a single system or in a balanced manner from both systems. Shown are UMAPs of the VAMP integration of the mouse-human dataset. (**b**) Entropy of the most probable prior assignment within every cell type or system (dots) corresponding to (**a**), with colors indicating runs with different prior initializations. Entropy was scaled by the maximal possible entropy.

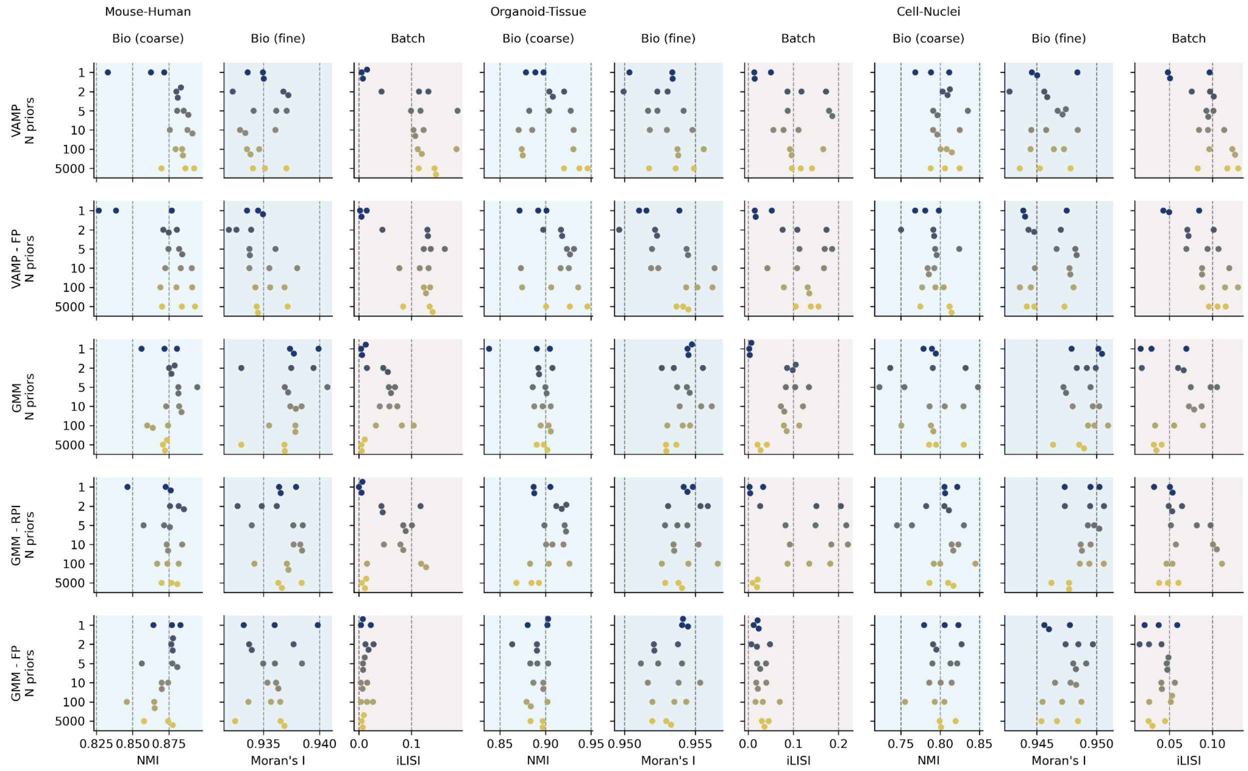

Supplementary Figure S8: Integration performance of VAMP and GMM with different prior component settings. Rows show different models and prior settings, including the number of prior components (y-axis values), columns show per-dataset integration metrics, and dots represent individual runs with different seeds for each setting. The displayed datasets are: pancreas mouse-human, retina organoid-tissue, adipose cell-nuclei. FP - fixed prior, RPI - random prior initialization.

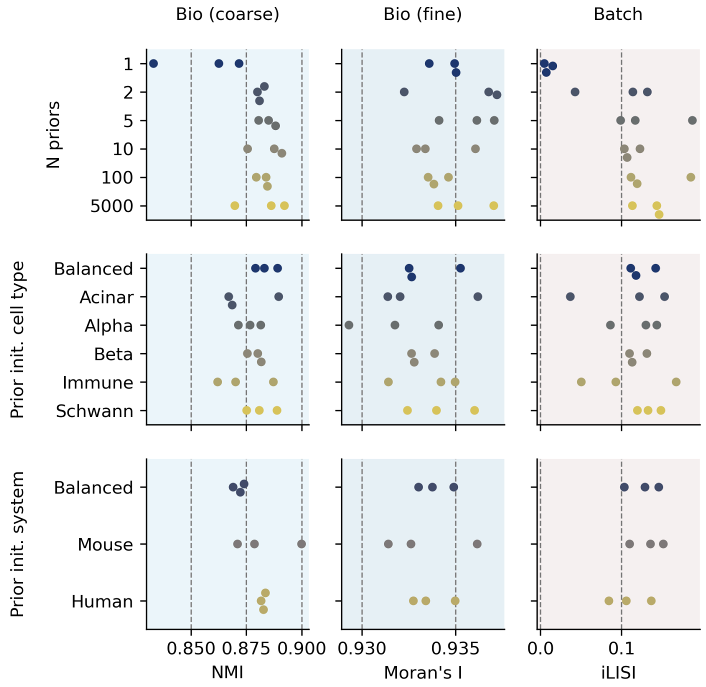

Supplementary Figure S9: Initializing pseudoinputs from all cell types or both systems or only one cell type or system does not have a major effect on the integration performance. Rows show different prior initializations, with the top row having random initialization with a different number of prior components (y-axis), and the other two rows being initialized with either one prior component sampled from each cell type or system ("Balanced") or from a single cell type or system with the number of prior components equal to the number of cell types (N=17) or systems (N=2), respectively. Columns show integration metrics for the pancreatic mouse-human dataset and dots represent individual runs with different seeds.

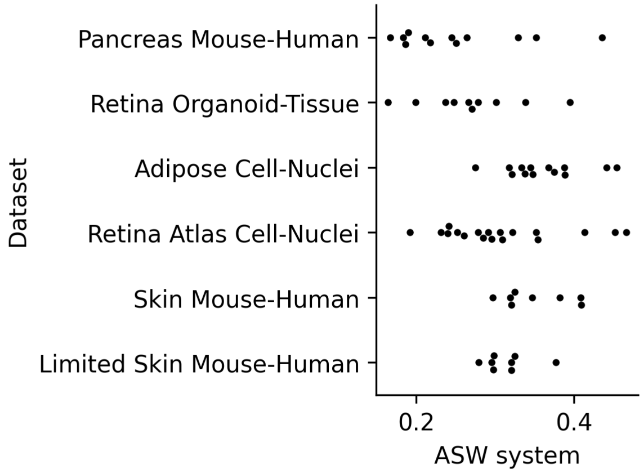

Supplementary Figure S10: Between-system batch effects strength prior to integration. Shown are ASW batch values computed with systems as batch covariates for individual cell types (points) in every dataset, with higher scores indicating stronger system mixing.

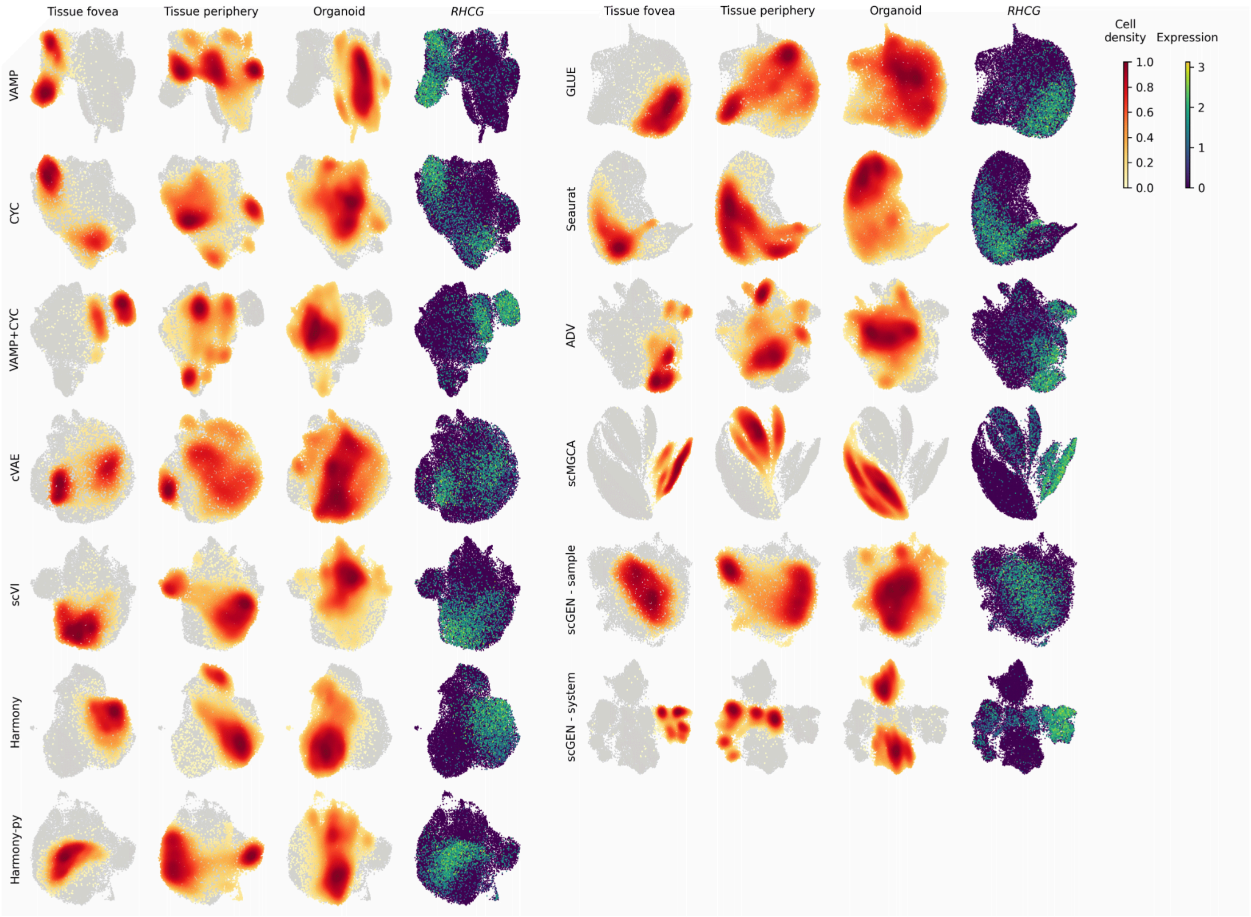

Supplementary Figure S11: Sample alignment in integrated Mueller cells. Plotted are UMAPs of Mueller cell subset from the retinal organoid-tissue dataset based on representative runs for the best hyperparameter setting of every model. We also added two example runs for scGEN with samples or systems as batch covariates. The UMAPs are colored by cell density in organoid and tissue cell groups and expression of foveal marker *RHCG*.

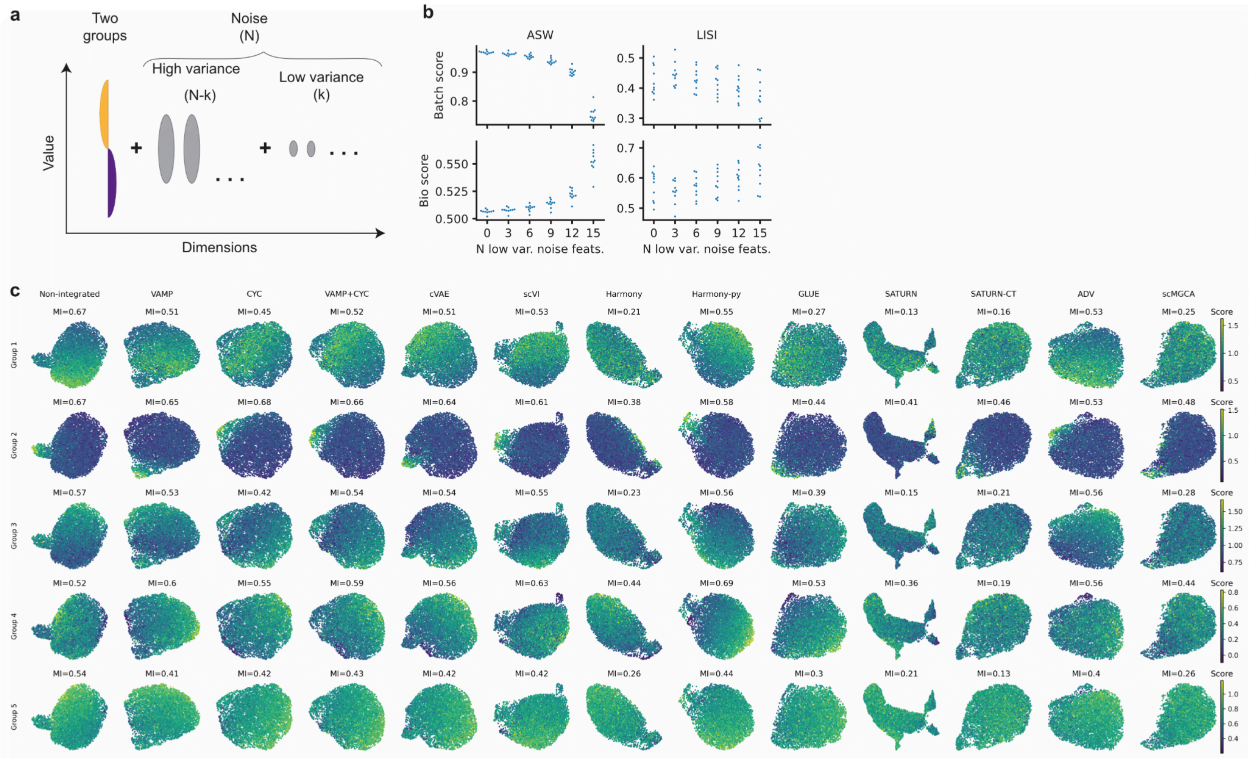

Supplementary Figure S12: Evaluation of integration metrics. (**a**) Overview of the data simulation process used for comparing distance and graph-based metrics. (**b**) Graph-based metrics are less affected by the value range of the noise dimensions than distance-based metrics. Shown are biological preservation and batch correction scores based on ASW or LISI when 0 to 15 of the noise dimensions are shrunk to have smaller variance. (**c**) Examples of Moran's I scores and corresponding expression patterns on UMAPs for five gene groups known to be variable in beta cells of healthy adult mice. For every model, one representative run from the best hyperparameter setting was selected (as shown in **Figure 5b**) and cells were subsetted to beta cells of one healthy adult mouse pancreatic sample, used for computing UMAP and Moran's I.

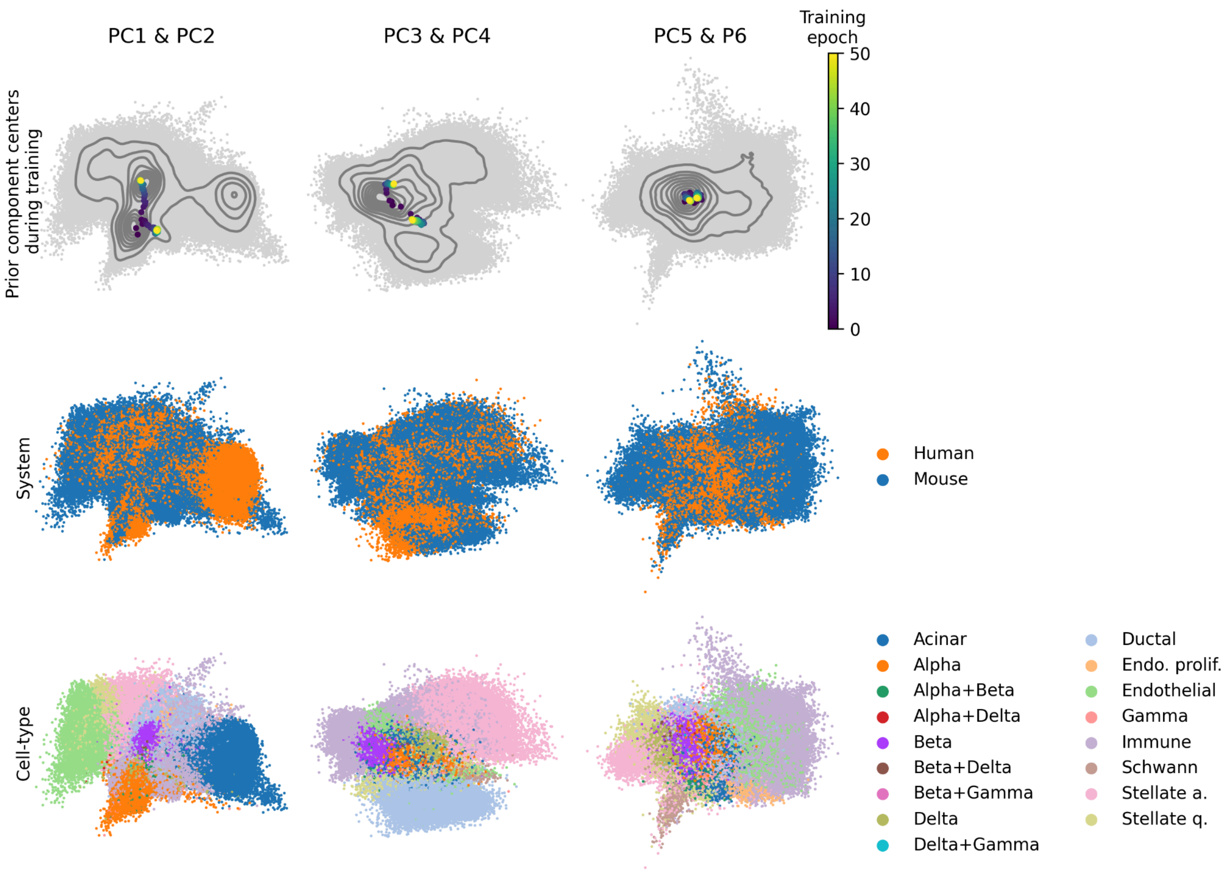

Supplementary Figure S13: The update of the VampPrior components during the training. The top row shows how the VampPrior components' (N=2) mean parameters (colored) are updated during the training in the space of the first six PCs (pairwise plots in columns) computed on the model's latent space. The contour lines represent the density of the input cells (every 10th quantile) and the gray points individual cells. The middle row shows the system covariate and the bottom row shows the cell type. Shown are UMAPs of VAMP integration of the pancreatic mouse-human dataset.

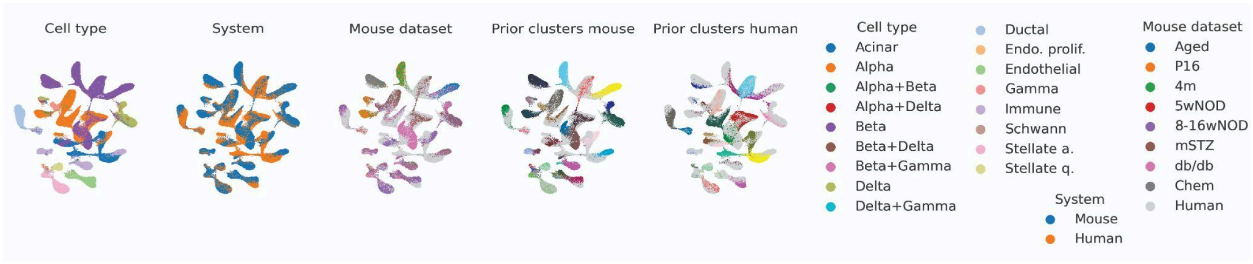

Supplementary Figure S14: Technical artifacts and errors in prior cluster labels are reflected in the final SATURN integration. The UMAPs display the per-system prior clusters and the dataset covariate for the mouse system. They were computed on a representative run from the best hyperparameter setting for SATUR integration of pancreatic mouse-human data. Prior cluster color legend is not shown as individual cluster names are not meaningful. For the mouse-specific covariates (datasets and clusters) the human data is shown as a background in light gray, and vice versa for the human covariates.

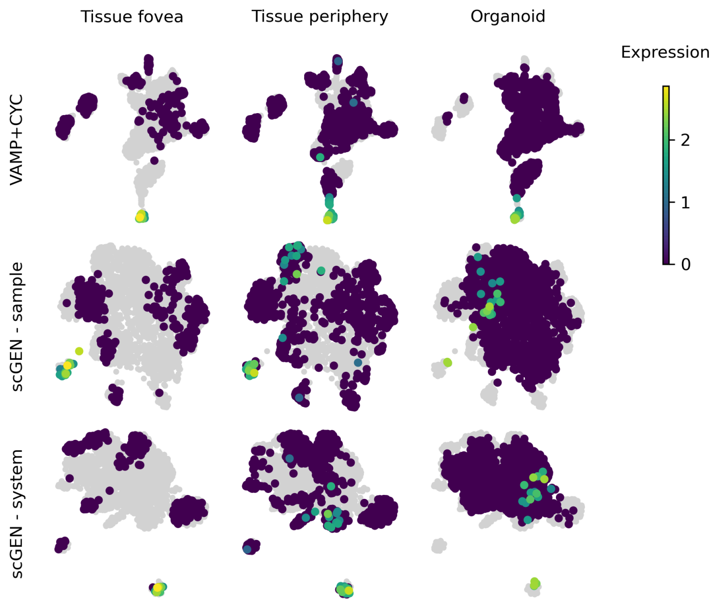

Supplementary Figure S15: Cell subtype preservation in amacrine cells after integration. The UMAPs are colored by expression of starburst amacrine cell marker *SLC18A3*. They were computed for the amacrine cell subset from the retinal organoid-tissue dataset based on a representative run for the best hyperparameter setting of VAMP+CYC and on two example runs of scGEN with samples or systems as batch covariates.

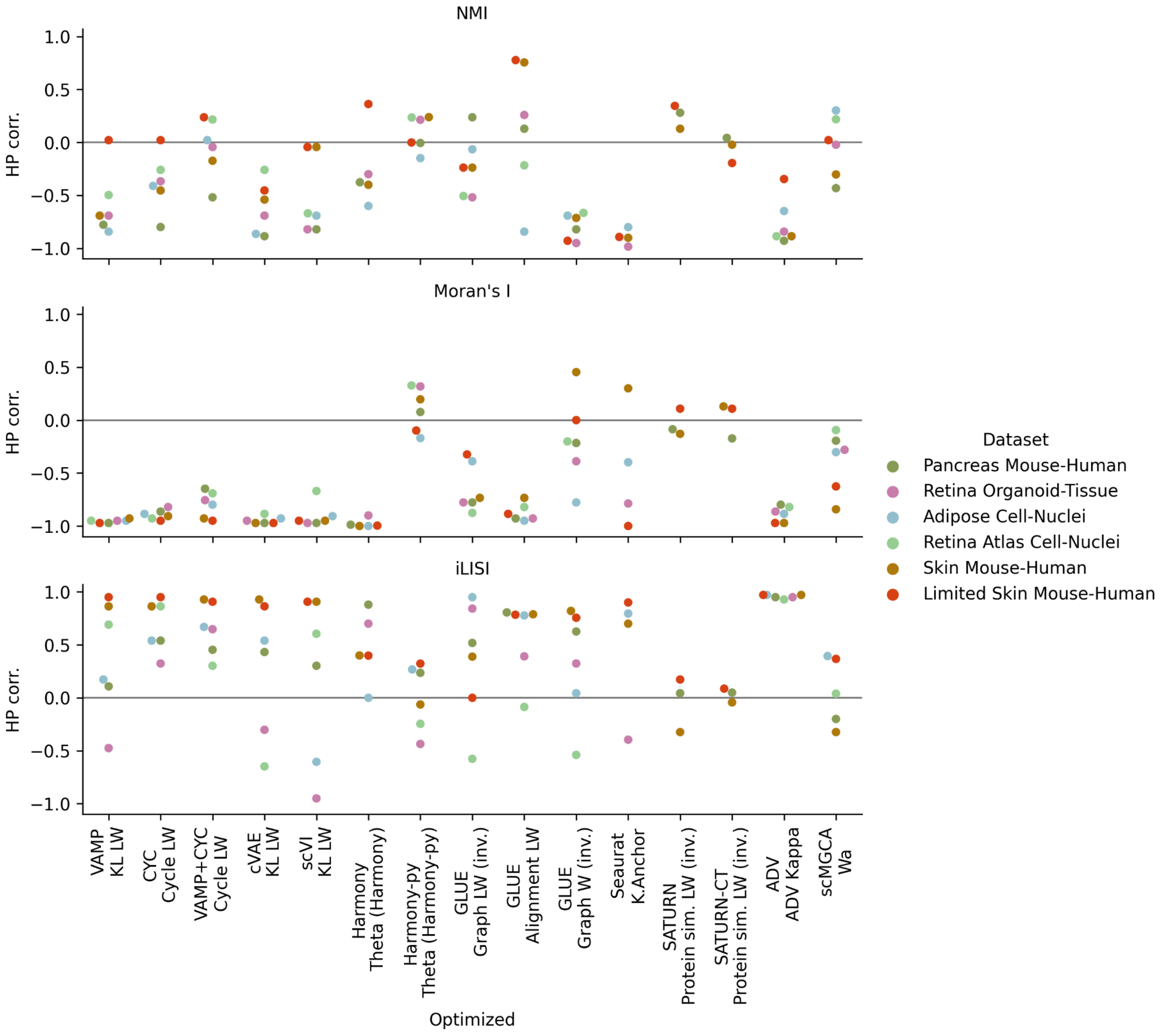

Supplementary Figure S16: Spearman correlation between hyperparameter values and integration metrics. Correlation (corr.) was computed for different combinations of models and tuned hyperparameters (x-axis) across all runs and hyperparameter values (HP) in the OTO setting as shown in **Supplementary Figure S1**, separately for each dataset. Values of hyperparameters that are expected to be negatively rather than positively associated with batch correction strength were inverted (inv.) before computing correlation.

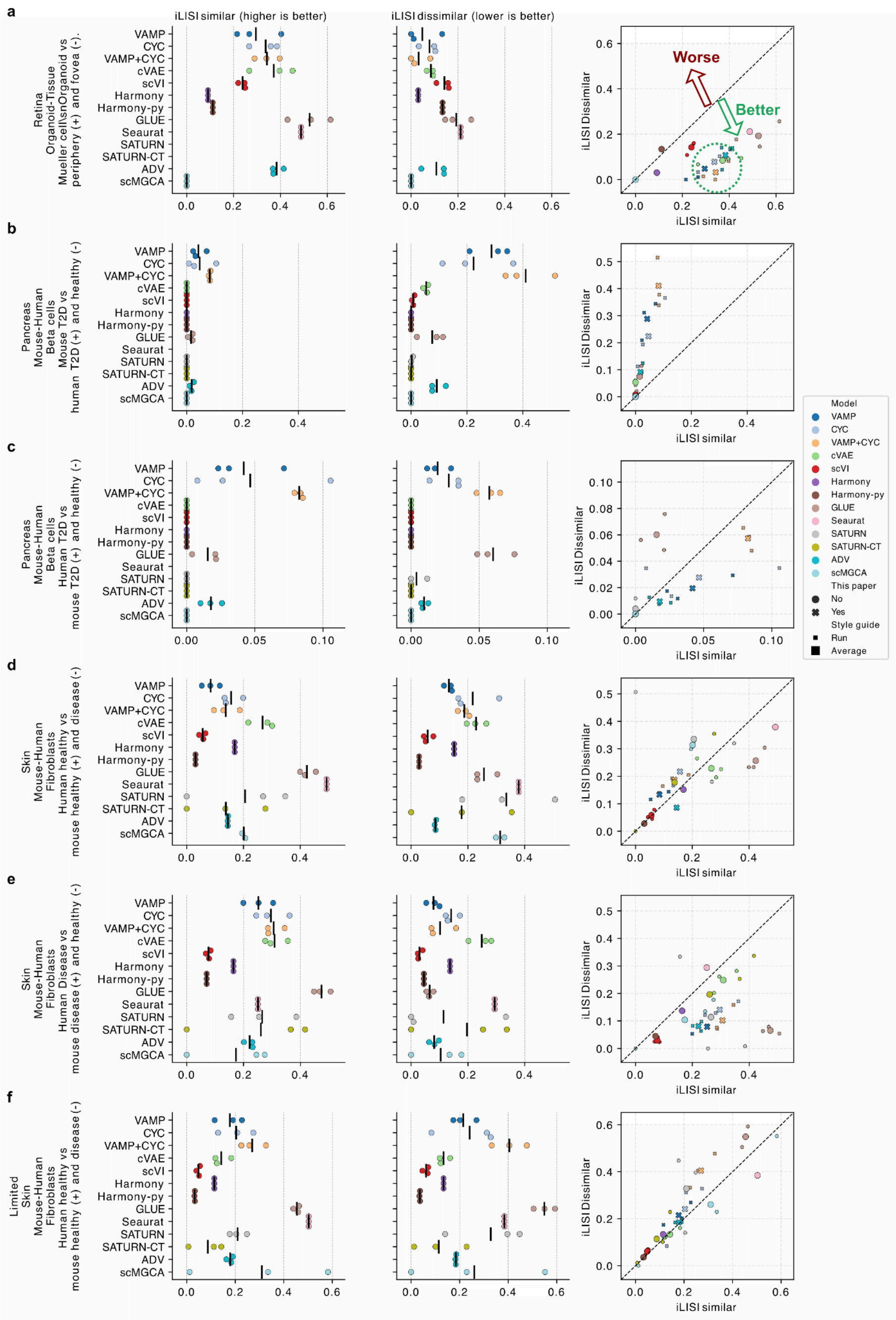

Supplementary Figure S17: Post-integration alignment of biologically related and unrelated conditions across systems. We show the alignment performance of individual models in terms of iLISI between similar biological conditions (such as disease in system 1 and disease in system 2) and iLISI between dissimilar biological conditions (such as disease in system 1 and healthy in system 2) using the best hyperparameter settings, with the mentioned scores in columns (higher iLISI values for similar conditions and lower iLISI values for dissimilar conditions indicate better integration performance) and cross-system data cases in rows, showing the average performance (vertical line) of three runs (dots). An example of a favorable matching of similar states with correct separation of dissimilar states is marked with a green dotted circle. The results of all hyperparameter settings are in **Supplementary Figure S18**.

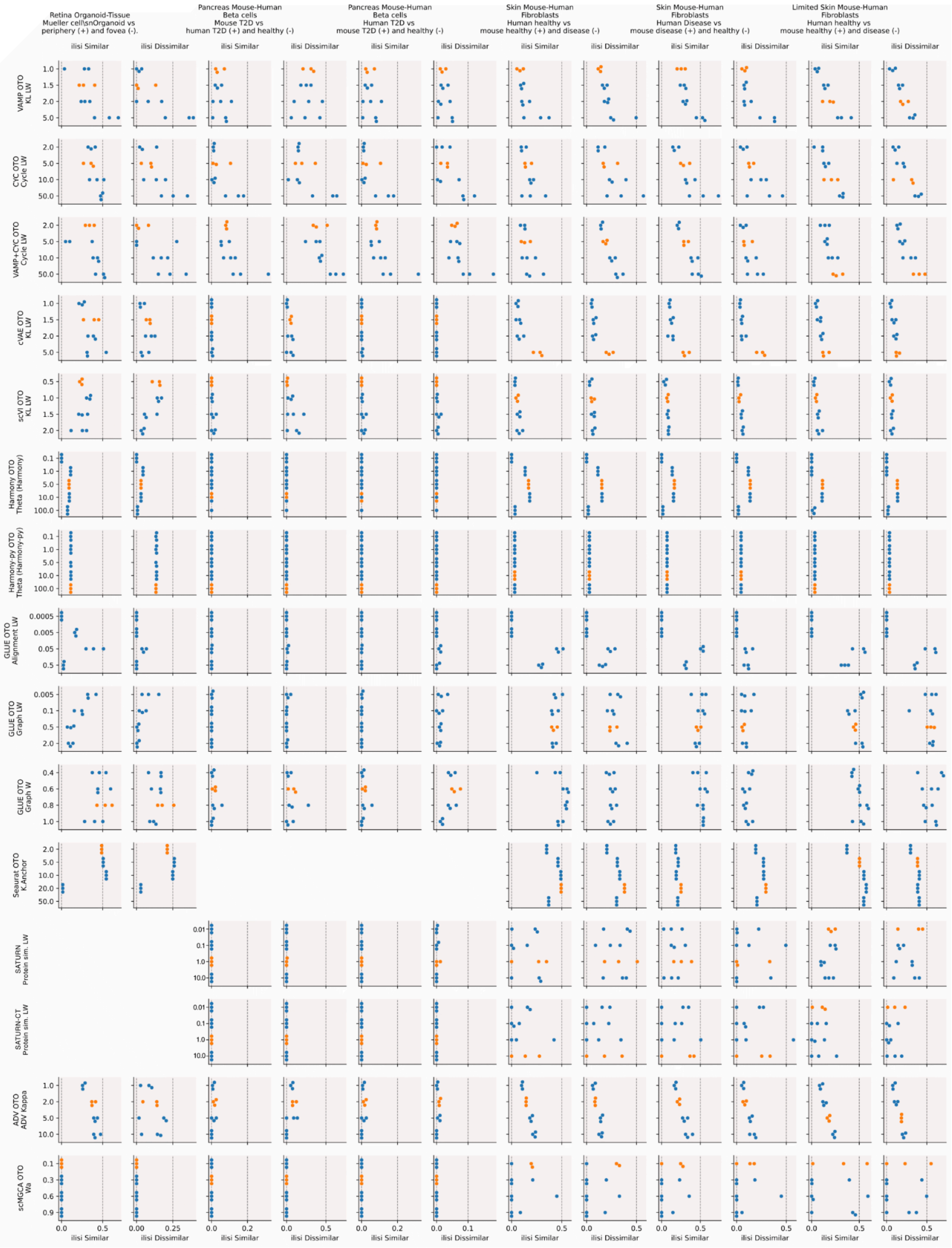
Retina Organoid-Tissue Mueller cell\snOrganoid vs periphery (+) and fovea (-).
Pancreas Mouse-Human Beta cells Mouse T2D vs human T2D (+) and healthy (-)
Pancreas Mouse-Human Beta cells Human T2D vs mouse T2D (+) and healthy (-)
Skin Mouse-Human Fibroblasts Human healthy vs mouse healthy (+) and disease (-)
Skin Mouse-Human Fibroblasts Human Disease vs mouse disease (+) and healthy (-)
Limited Skin Mouse-Human Fibroblasts Human healthy vs mouse healthy (+) and disease (-)
ilisi Similar
ilisi Dissimilar
VAMP OTO KL LW
CYC OTO Cycle LW
VAMP+CYC OTO Cycle LW
cVAE OTO KL LW
scVI OTO KL LW
Harmony OTO Theta (Harmony)
Harmony-py OTO Theta (Harmony-py)
GLUE OTO Alignment LW
GLUE OTO Graph LW
GLUE OTO Graph W
Seaurat OTO K.Anchor
SATURN Protein sim. LW
SATURN-CT Protein sim. LW
ADV OTO ADV Kappa
scMGCA OTO Wa

Supplementary Figure S18: The performance of aligning biological conditions after integration across models and different model hyperparameters. Rows show different models and tested hyperparameters (y-axis values), columns show mixing scores (iLISI) between similar states and dissimilar states, and dots represent individual runs with different seeds. For each setting, the top runs are marked in orange.

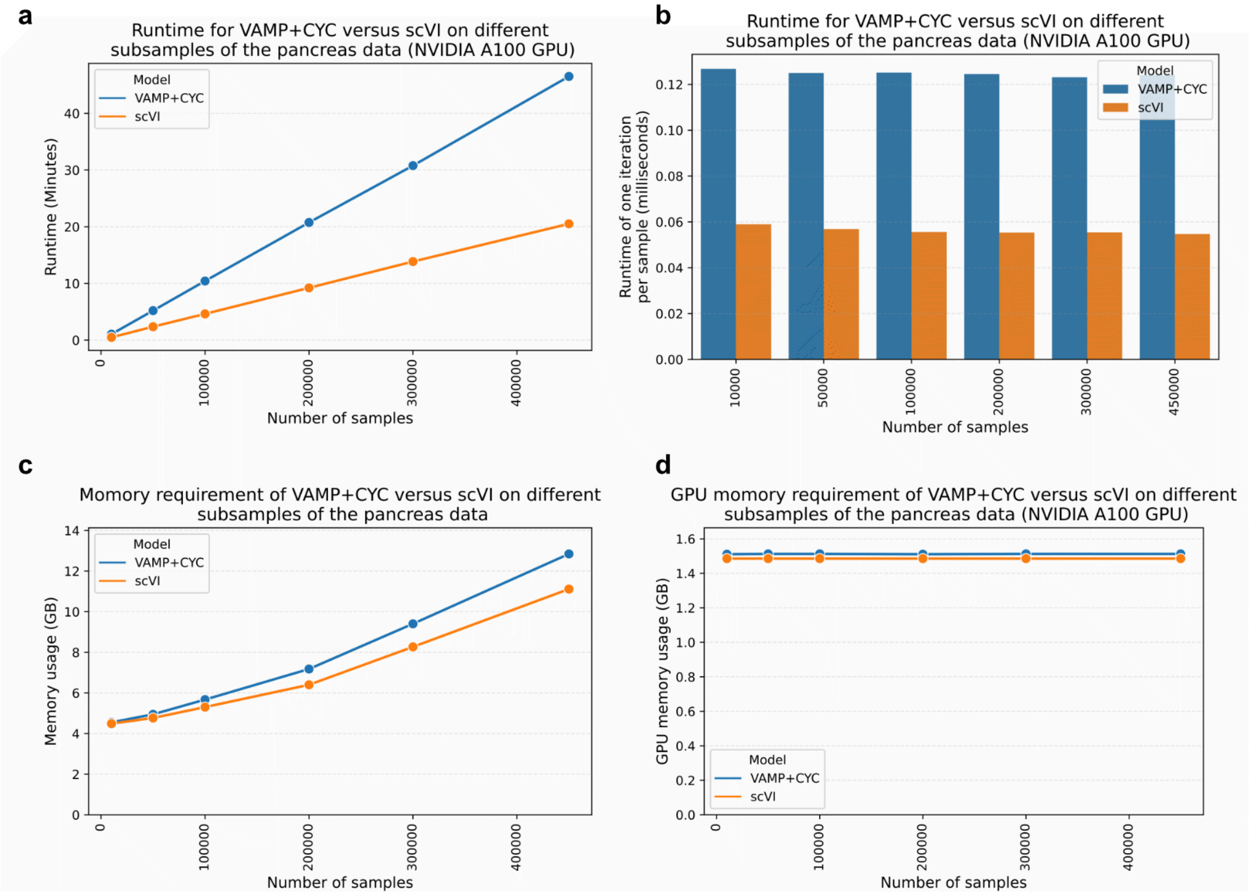

Supplementary Figure S19: Use of computational resources by VAMP+CYC and scVI. (**a**) Runtime is shown for 50 epochs for all dataset sizes. In practice, runtime on large datasets can be further reduced as fewer epochs are required due to a larger number of within-epoch steps. This is standard practice for cVAE models in the well-established scvi-tools package, which also contains scVI and implementation of our VAMP+CYC model (sysVI). (**b**) The runtime per sample iteration remains constant regardless of dataset size, as expected. (c) Memory and (d) GPU memory requirement are shown for the same setup. The required amount of memory and GPU memory in VAMP+CYC is comparable to that of scVI.

Supplementary tables

Supplementary Table S1: Comparison of pre-integration sample distances within and between systems. One-sided Mann–Whitney U test was used to compare distributions of per-cell-type sample distances in the PCA space within a system (control) and between systems (case) for every dataset (sheet names). Column names: cell_type - tested cell type; system - control group, with "within/between" in the mouse-human data referring to within/between dataset distances in the mouse data; u - test statistic; padj - adjusted p-value; n_system and n_crossystem - number of distances (observations) in the control and case groups, respectively.

Supplementary Table S2: Statistical comparison of model performance on different datasets. Welch's t-test was used to compare integration performance between different models in individual datasets. For each model, the best hyperparameter setting was selected as described in the methods and shown in **Figure 5**. Column names: dataset - the integrated dataset, metric - integration metric, model - the used integration model, p - p-value, t - test statistic, padj_fdr_tsbh - adjusted p-value, model_cond - model used as the test condition, model_ctrl - model used as the control condition, higher - model with higher value of the tested metric.
